# Identification of myeloid-derived growth factor as a mechanically-induced, growth-promoting angiocrine signal for human hepatocytes

**DOI:** 10.1038/s41467-024-44760-y

**Published:** 2024-02-05

**Authors:** Linda Große-Segerath, Paula Follert, Kristina Behnke, Julia Ettich, Tobias Buschmann, Philip Kirschner, Sonja Hartwig, Stefan Lehr, Mortimer Korf-Klingebiel, Daniel Eberhard, Nadja Lehwald-Tywuschik, Hadi Al-Hasani, Wolfram Trudo Knoefel, Stefan Heinrich, Bodo Levkau, Kai C. Wollert, Jürgen Scheller, Eckhard Lammert

**Affiliations:** 1 Heinrich Heine University Düsseldorf, Faculty of Mathematics and Natural Sciences, Institute of Metabolic Physiology, 40225 Düsseldorf, Germany; 2 Institute for Vascular and Islet Cell Biology, German Diabetes Center (DDZ), Leibniz Center for Diabetes Research at Heinrich Heine University, 40225 Düsseldorf, Germany; 3 German Center for Diabetes Research (DZD e.V.), Helmholtz Zentrum München, 85764 Neuherberg, Germany; 4 Institute of Biochemistry and Molecular Biology II, Medical Faculty and University Hospital Düsseldorf, Heinrich Heine University Düsseldorf, 40225 Düsseldorf, Germany; 5 Institute for Molecular Medicine III, Medical Faculty and University Hospital Düsseldorf, Heinrich Heine University Düsseldorf, 40225 Düsseldorf, Germany; 6 Institute for Clinical Biochemistry and Pathobiochemistry, German Diabetes Center (DDZ), Leibniz Center for Diabetes Research at Heinrich Heine University, Medical Faculty, 40225 Düsseldorf, Germany; 7 Division of Molecular and Translational Cardiology, Department of Cardiology and Angiology, Hannover Medical School, 30625 Hannover, Germany; 8 Department of General, Visceral, Thorax and Pediatric Surgery, Medical Faculty and University Hospital Düsseldorf, Heinrich Heine University Düsseldorf, 40225 Düsseldorf, Germany; 9 Department of General, Visceral and Transplantation Surgery, University Hospital Center Mainz, 55131 Mainz, Germany

**Keywords:** Hepatocytes, Physiology

## Abstract

Recently, we have shown that after partial hepatectomy (PHx), an increased hepatic blood flow initiates liver growth in mice by vasodilation and mechanically-triggered release of angiocrine signals. Here, we use mass spectrometry to identify a mechanically-induced angiocrine signal in human hepatic endothelial cells, that is, myeloid-derived growth factor (MYDGF). We show that it induces proliferation and promotes survival of primary human hepatocytes derived from different donors in two-dimensional cell culture, via activation of mitogen-activated protein kinase (MAPK) and signal transducer and activator of transcription 3 (STAT3). MYDGF also enhances proliferation of human hepatocytes in three-dimensional organoids. In vivo, genetic deletion of MYDGF decreases hepatocyte proliferation in the regenerating mouse liver after PHx; conversely, adeno-associated viral delivery of MYDGF increases hepatocyte proliferation and MAPK signaling after PHx. We conclude that MYDGF represents a mechanically-induced angiocrine signal and that it triggers growth of, and provides protection to, primary mouse and human hepatocytes.

## Abstract (web summary)

After partial liver resection, the remaining liver blood vessels receive more blood flow and get mechanically stretched. Here the authors show that MYDGF is released from the stretched cells of these liver vessels, and that it is required and sufficient to promote liver regeneration and cell proliferation.

## Introduction

Liver regeneration is the unique process of the liver to fully restore its original size, in particular after partial hepatectomy (PHx) in mice and humans^1–3^. Shortly after PHx, the hepatic blood vessels experience increased shear stress due to more blood flowing through the remaining part of the liver than before the PHx^4^. This shear stress induces vasodilation and concomitantly mechanical stretching of the endothelial cells (ECs) within the small hepatic blood vessels. Shortly after these biomechanical changes, liver regeneration starts with a robust initiation of hepatocyte proliferation^5–7^.

Mechanical stretching of hepatic ECs, as seen during shear stress-induced vasodilation after PHx^8^, can be partially mimicked in vitro using an oscillatory cell stretching device. Notably, upon this oscillatory mechanical stretching, primary human hepatic ECs were shown to release growth-promoting, cell-protective angiocrine signals^5^, known to be required for proper liver regeneration. In general, angiocrine signals (including ATP, which activates purinergic signaling)^9–11^ were demonstrated by multiple different laboratories to be needed for liver regeneration and to regulate hepatocyte proliferation and angiogenesis^8,11–19^. In vivo, several studies have shown that angiocrine signals, including hepatocyte growth factor (HGF), epidermal growth factor (EGF), interleukin-6 (IL-6) and tumor necrosis factor-α (TNF-α), stimulate mouse hepatocyte proliferation^7,20–22^. Although hepatocytes have an enormous capacity to regenerate and proliferate in vivo in mice and humans (in particular after PHx), to our knowledge, not a single angiocrine signal, other than HGF, has been identified yet that alone stimulates proliferation of human hepatocytes in vitro or ex vivo^23,24^. However, perfusion of isolated human livers has been shown to protect human hepatocytes and increase success of subsequent liver transplantation, indicating that in the human liver (like the mouse liver), perfusion might be key to turn on potent, cell-protective mechanisms^25,26^. To better understand the underlying molecular mechanisms of liver regeneration, growth and protection, there is an urgent need to identify angiocrine signals that can promote hepatocyte proliferation and protect them from cell death, in particular in the human setting.

In this work, we used a mass spectrometry-based approach with the aim to identify angiocrine signals capable of promoting human hepatocyte proliferation. We identified myeloid-derived growth factor (MYDGF), a protein previously identified in a secretome screen on human bone marrow cells and characterized in the context of myocardial infarct healing in mice and humans^27–30^, as a mechanically-induced angiocrine signal. We further showed that MYDGF had growth-promoting and cell-protective effects on primary human hepatocytes and that it was required and sufficient to enhance hepatocyte proliferation in mice after PHx.

## Results

### Mass spectrometric approach to identify angiocrine signals induced by mechanical stimulation of human hepatic endothelial cells

**Fig. 1:**
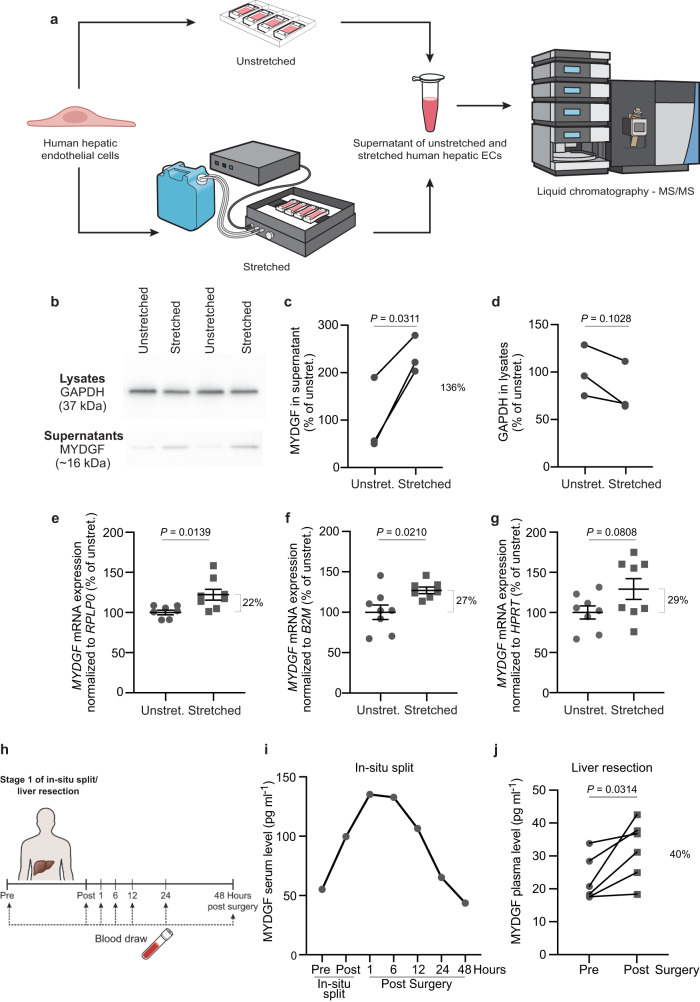
Identification of MYDGF as a mechanically-induced angiocrine signal. **a** Experimental design for identification of angiocrine signals. Primary human hepatic endothelial cells (ECs) were mechanically stretched for 1.5 h (20% static stretch for 30 min plus 20% cyclic stretch (30 cycles/min) for 1 h), and the supernatants of unstretched and stretched hepatic ECs were analyzed by liquid chromatography tandem-mass spectrometry (LC-MS/MS). Myeloid-derived growth factor (MYDGF) was identified in the supernatants of mechanically stimulated hepatic ECs. **b** Representative Western blot images of supernatants (lower band) and lysates (upper band) of unstretched and stretched human hepatic ECs showing MYDGF (~16 kilodalton (kDa)) and GAPDH (37 kDa). Quantification of MYDGF levels in supernatants (**c**) and GAPDH levels in lysates (**d**) of unstretched and stretched hepatic ECs, shown as percentage of unstretched conditions; *n* = 3 independent experiments with 7 stretch chambers on average each. Two of the three independent experiments were performed with a male, 52-year-old human hepatic EC donor and one experiment with a 27-year-old female human hepatic EC donor. Quantification of *MYDGF* mRNA expression levels normalized to three housekeeping genes, i.e., *RPLP0* (**e**), *B2M* (**f**) and *HPRT* (**g**) in lysates of unstretched and stretched hepatic ECs. *n* = 7 unstretched versus *n* = 8 stretched chambers (**e**), *n* = 8 unstretched versus *n* = 7 stretched chambers (**f**), *n* = 8 unstretched versus *n* = 8 stretched chambers (**g**). Primary human hepatic EC donor: male, 52 years. **h** Timeline of blood draw in a human patient pre and post in-situ split liver surgery. **i** MYDGF serum levels in a human patient at different timepoints (stage 1 of in situ-split/ liver resection); *n* = 1 patient (male, 70–80-year-old). **j** MYDGF plasma levels in six human patients pre and post liver resection (males, 54–89-year-old, body mass index (BMI) < 30 kg m^-2^). Data are presented as mean ± SEM (**e**–**g**). *P* values were calculated using two-tailed paired Student’s *t*-test (**c**, **d**, **j**) and unpaired (**e**–**g**) Student’s *t*-test with Welch’s correction. Source data are provided as a Source Data file.

After PHx, hepatic blood vessels encounter an increased shear stress-induced vasodilation that exposes the hepatic ECs to mechanical stretching, which triggers the release of growth-promoting angiocrine signals^4,5,14^. To identify signals that promote hepatocyte growth, we analyzed the supernatants of unstretched and stretched primary human hepatic ECs by liquid chromatography tandem-mass spectrometry (LC-MS/MS, Fig. 1).

Therefore, human hepatic ECs were plated in elastic silicone chambers and were either not stretched (unstretched) or mechanically stretched using an automated cell stretching device (Fig. 1a). After 1.5 h with and without mechanical stimulation, the supernatants of the hepatic ECs were collected and analyzed via LC-MS/MS. We identified 70 proteins in the supernatants of unstretched and stretched human hepatic ECs (Supplementary Table 1). To ensure that stretching did not cause cell death and resulted in false hits, we checked the viability of unstretched and mechanically stretched hepatic ECs. We detected slightly fewer dead cells in the stretched condition than in the unstretched condition (Supplementary Fig. 1). Notably, 22 proteins were identified exclusively in the supernatants of mechanically-stimulated hepatic ECs; and six of these proteins were known secreted proteins. Myeloid-derived growth factor (MYDGF) appeared as the first hit in the LC-MS/MS list of the latter proteins, and besides MYDGF, galectin-3, nicotinamide phosphoribosyltransferase, epididymal secretory protein E1, glycine--tRNA ligase and protein-glutamine gamma-glutamyltransferase 2 were identified. Notably, all of these proteins have been previously cited in some reports on the liver^31–35^.

We first checked whether the growth factor MYDGF was indeed secreted by primary human hepatic ECs. In Western blot experiments, MYDGF levels were found to be higher in supernatants of mechanically-stretched compared to unstretched hepatic ECs, whereas GAPDH protein levels did not change significantly in cell lysates (Fig. 1b–d). The mRNA expression of *MYDGF* was also higher in lysates derived from mechanically-stretched compared to unstretched hepatic ECs (Fig. 1e–g). To find out whether MYDGF is released into the blood stream after initiation of human liver regeneration, we first analyzed the serum of a male patient with a metastatic colorectal carcinoma pre and post stage 1 in-situ split liver surgery (Fig. 1h, i). This particular surgery splits the liver into a healthy and a diseased part (i.e., the one with the cancer metastases), and it reduces the blood flow to the diseased part, while it increases the blood flow to the healthy part of the liver^36^. The surgery results in vasodilation (and endothelial cell stretching) with a concomitant growth of the healthy part of the liver (stage 1), so that it reaches a size large enough to fulfill the body’s demand for liver function. The increase in size of the healthy liver allows the surgeon to subsequently remove the diseased part from the split liver (stage 2)^36^. Notably, the concentration of MYDGF increased right after stage 1 surgery and was elevated in the serum during the following 12 h, before MYDGF serum concentrations declined to pre-operation level (Fig. 1i). To analyze more patients, blood samples were analyzed from 6 patients with hepatocellular carcinoma (HCC) undergoing liver resection (Fig. 1j), again representing a stimulus for growth of the remaining healthy liver. During this surgery, the healthy part of the liver was large enough, so that the diseased part of the liver could be directly removed by the surgeon. We found that MYDGF concentrations increased in the plasma of these patients by approximately 40% after surgery (Fig. 1j). Thus, using independent methods, we could demonstrate that MYDGF (known in the heart to be secreted from bone-marrow derived monocytes and macrophages^27^) is (i) an angiocrine signal secreted by primary human hepatic ECs in response to mechanical stimulation and (ii) increased in blood samples from human patients who underwent liver surgery known to induce liver regeneration.

### MYDGF promotes proliferation and prevents apoptosis of human hepatocytes derived from different human donors

**Fig. 2:**
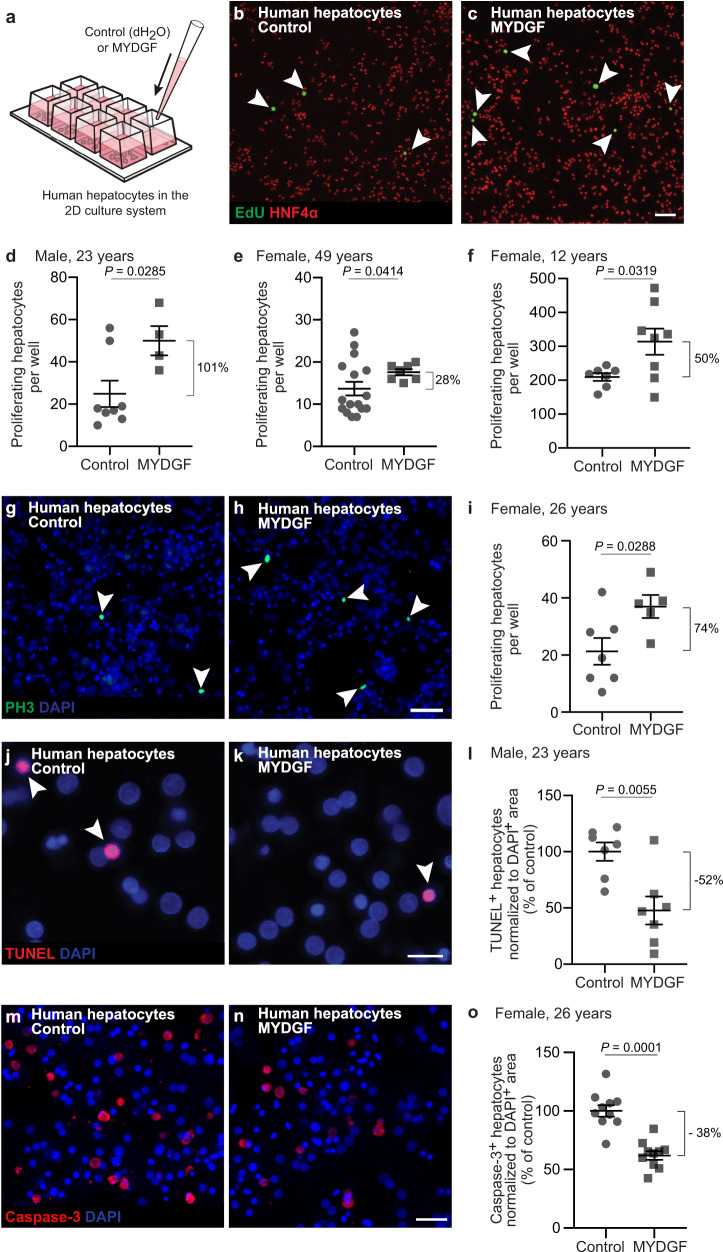
MYDGF stimulates proliferation and prevents apoptosis of primary human hepatocytes in vitro. **a** Schematic illustration of how human hepatocytes were treated with recombinant myeloid-derived growth factor (MYDGF). Representative laser scanning microscopy (LSM) images of human hepatocytes treated without (**b**) or with (**c**) recombinant MYDGF. White arrowheads point to proliferating cells stained for 5-ethynyl-2’-deoxyuridine (EdU, green); hepatocytes stained for hepatocyte nuclear factor 4α (HNF4α, red). Quantification of proliferating hepatocytes from three different human donors: (**d**) male, 23 years, *n* = 8 wells and *n* = 4 wells treated without or with MYDGF, respectively; (**e**) female, 49 years, *n* = 16 wells and *n* = 7 wells treated without or with MYDGF, respectively; (**f**) female, 12 years, *n* = 7 wells and *n* = 8 wells treated without or with MYDGF, respectively. Representative LSM images of human hepatocytes treated without (**g**) or with (**h**) recombinant MYDGF, stained for the proliferation marker phospho-Histone H3 (PH3, green). Cell nuclei were counterstained for DAPI (blue). **i** Quantification of proliferating human hepatocytes from a female, 26-year-old donor; *n* = 7 wells and *n* = 5 wells treated without or with MYDGF, respectively. Representative LSM images of human hepatocytes treated without (**j**) or with (**k**) recombinant MYDGF. Apoptotic cells were visualized by TUNEL (red) and cell nuclei were counterstained for DAPI (blue). **l** Quantification of TUNEL^+^ human hepatocytes from a male, 23-year-old donor; *n* = 7 wells each. Representative LSM images of human hepatocytes treated without (**m**) or with (**n**) recombinant MYDGF. Apoptotic cells were visualized by caspase-3 staining (red), and cell nuclei were counterstained for DAPI (blue). **o** Quantification of caspase-3^+^ human hepatocytes from a female, 26-year-old donor; *n* = 10 wells each. Scale bars: 100 µm (**c**), 50 µm (**h**, **n**), 20 µm (**k**). Data are presented as mean ± SEM. *P* values were calculated using two-tailed unpaired Student’s *t*-test with Welch’s correction. Source data are provided as a Source Data file.

To determine whether MYDGF is a growth-promoting angiocrine signal, we next tested its effect on primary human hepatocytes (Fig. 2). More specifically, we treated in vitro cultivated human hepatocytes from different human donors with recombinant human MYDGF (Fig. 2a), and we subsequently stained for the proliferation markers 5-ethynyl-2’-deoxyuridine (EdU, Fig. 2b–f and Supplementary Fig. 2a–c) and phospho-Histone H3 (PH3, Fig. 2g–i and Supplementary Fig. 2d–f). Notably, treatment with a MYDGF concentration as low as 1 ng/ml was observed to significantly promote proliferation of two-dimensionally (2D) cultured primary human hepatocytes (Fig. 2d–f, Fig. 2i and Supplementary Fig. 2c, f), with the exception of hepatocytes from a drug-dependent 49-year-old donor where 5 ng/ml MYDGF was needed to induce proliferation (Fig. 2e). In contrast, MYDGF did not induce proliferation of the cancer cell line HepG2 (Supplementary Fig. 2g–i), possibly because this immortalized cell line did not reflect the situation of adult human hepatocytes (Supplementary Fig. 2j).

Next, we tested another mechanical stretch-induced angiocrine signal identified in the mass spectrometric screen, i.e., galectin-3, as well as a mechanically unresponsive angiocrine signal, i.e., connective tissue growth factor (CTGF). However, none of these proteins were found to induce proliferation of human hepatocytes (Supplementary Fig. 3). Therefore, we continued to work on MYDGF and tested next whether it influenced the viability of human hepatocytes. Treatment with MYDGF was found to prevent apoptosis of human hepatocytes (Fig. 2j–o and Supplementary Fig. 2k–q), thus indicating a net positive effect of MYDGF on primary human hepatocytes.

**Fig. 3:**
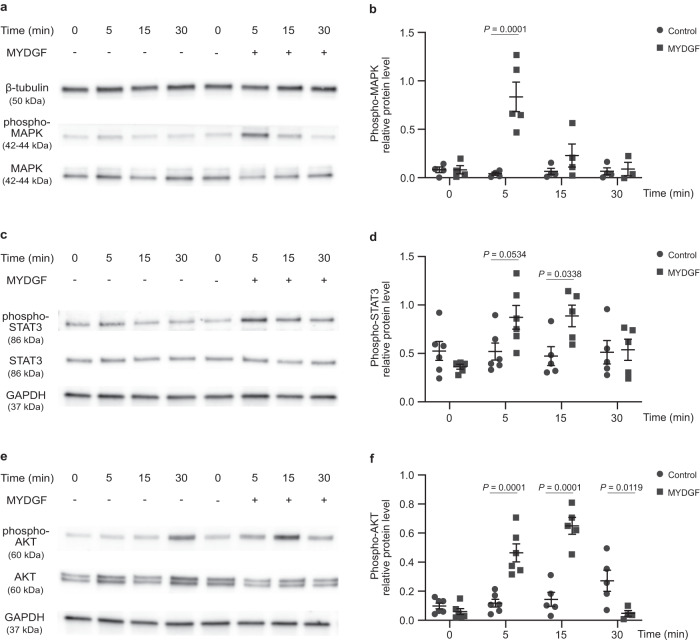
MYDGF activates phosphorylation of MAPK, STAT3 and AKT. Human hepatocytes were treated without or with recombinant myeloid-derived growth factor (MYDGF) for different times (0, 5, 15 and 30 min). **a** Western blots for phosphorylated mitogen-activated protein kinase (phospho-MAPK, 42–44 kDa; T202/Y204), MAPK (42–44 kDa) and β-tubulin (50 kDa) in lysates from human hepatocytes. **b** Quantification of phospho-MAPK protein levels normalized to MAPK and β-tubulin protein levels. *n* = 4 (0, 5, 15 and 30 min) control- versus *n* = 4 (0 and 15 min), *n* = 5 (5 min) and *n* = 3 (30 min) MYDGF-treated human hepatocytes. **c** Western blots for phosphorylated signal transducer and activator of transcription 3 (phospho-STAT3, 86 kDa; S727), STAT3 (86 kDa) and GAPDH (37 kDa). **d** Quantification of phospho-STAT3 protein levels normalized to STAT3 and GAPDH protein levels. *n* = 6 (0 and 5 min) and *n* = 5 (15 and 30 min) control- versus *n* = 5 (0, 15 and 30 min) and *n* = 6 (5 min) MYDGF-treated human hepatocytes. **e** Western blots for phospho-AKT (60 kDa; S473), AKT (60 kDa) and GAPDH (37 kDa). **f** Quantification of phospho-AKT protein levels normalized to AKT and GAPDH protein levels. *n* = 6 (0 and 5 min) and *n* = 5 (15 and 30 min) control- versus *n* = 6 (0 and 5 min), *n* = 5 (15 min) and *n* = 4 (30 min) MYDGF-treated human hepatocytes. Human hepatocyte donor: female, 26-year-old. Data are presented as mean ± SEM. *P* values were calculated using two-way ANOVA followed by Šidák´s post hoc test. Source data are provided as a Source Data file.

We then asked which signaling pathway was involved in the proliferative effect of MYDGF on human hepatocytes (Fig. 3). Previous studies suggested that MYDGF induced phosphorylation of mitogen-activated protein kinase (MAPK or more specifically, Erk1/2), signal transducer and activator of transcription 3 (STAT3) and AKT^27,37–40^. We transferred these findings to primary human hepatocytes and observed a rapid, transient increase in phosphorylation of MAPK (T202/Y204), STAT3 (S727) and AKT (S473), already 5 to 15 min after addition of MYDGF to the human hepatocytes (Fig. 3). To investigate whether activation of MAPK and STAT3 were involved in MYDGF-dependent hepatocyte proliferation, we inhibited phosphorylation of MAPK and STAT3 in human hepatocytes (Supplementary Fig. 4a–d). We observed a significantly decreased proliferation rate of hepatocytes derived from three human donors upon treatment with the inhibitors (Supplementary Fig. 4e–k). Thus, our screen led to the identification of a mechanically-induced angiocrine signal that promotes proliferation and survival of primary human hepatocytes and activates MAPK and STAT3, both of which are required for primary human hepatocyte proliferation.

### MYDGF promotes proliferation of three-dimensional human hepatocyte organoids

**Fig. 4:**
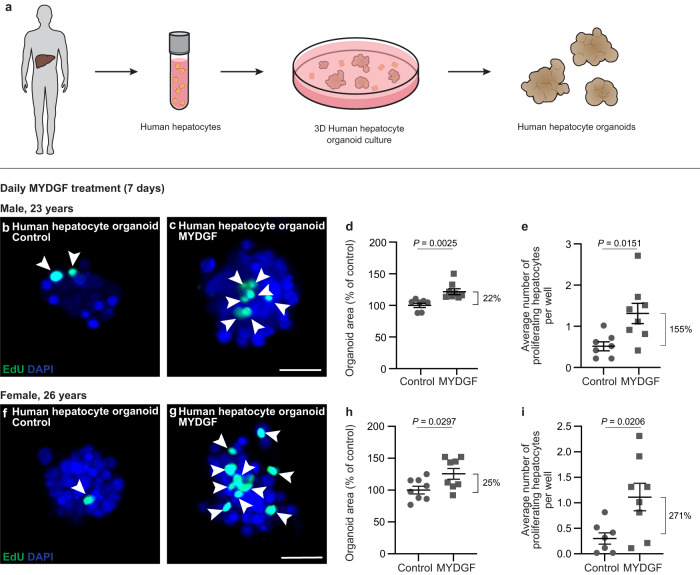
MYDGF enhances cell proliferation in human hepatocyte organoids. **a** Schematic illustration of growing 3D organoids from human hepatocytes. Hepatocytes isolated from human liver were cultured in cell culture plates with low adhesion. After 3 days, organoids were formed, which could subsequently be treated with recombinant myeloid-derived growth factor (MYDGF). Representative laser scanning microscopy (LSM) images (maximum intensity projections) of human hepatocyte organoids treated daily without (**b**) or with (**c**) recombinant MYDGF. Proliferating cells were stained for 5-ethynyl-2’-deoxyuridine (EdU, green) and cell nuclei counterstained for DAPI (blue). Quantification of human organoid area (**d**) and proliferating human hepatocytes per well (**e**) in control- versus MYDGF-treated human hepatocyte organoids. *n* = 7 versus *n* = 8 wells with 5 organoids each. Representative LSM images (maximum intensity projections) of human hepatocyte organoids treated daily without (**f**) or with (**g**) recombinant MYDGF. Proliferating cells were stained for EdU (green) and cell nuclei counterstained for DAPI (blue). Quantification of human organoid area (**h**) and proliferating human hepatocytes per well (**i**) in control-versus MYDGF-treated human hepatocyte organoids. *n* = 8 wells with 5 organoids each (**h**) and *n* = 7 versus *n* = 8 wells with 5 organoids each (**i**). Donors: male, 23 years (**b**–**e**), and female, 26 years (**f**–**i**). Scale bar: 50 µm (**c**, **g**). Data are presented as mean ± SEM. *P* values were calculated using two-tailed unpaired Student’s *t*-test with Welch’s correction. Source data are provided as a Source Data file.

In general, organoids are considered more physiologically representative than classic 2D cell cultures, since the cells of organoids are located within a three-dimensional (3D) environment with a higher number of cell-to-cell contacts^41^. Thus, to validate our findings in a physiological environment, we established 3D organoid cultures of primary human hepatocytes derived from male and female donors (Fig. 4). The organoids were cultured in a Matrigel suspension^42,43^; and after three days, grape-like and hepatocyte nuclear factor 4α (HNF4α)-positive human organoids formed (Fig. 4a and Supplementary Fig. 5a–c). We chose to treat the organoids with 10 ng/ml recombinant MYDGF, which is a concentration 10 times higher than in 2D cell culture (1 ng/ml), to ensure that sufficient MYDGF reached all hepatocytes in the 3D culture to exert its effects. The organoids treated with a single dose of 10 ng/ml MYDGF showed an increased organoid area, and a numerical increase of 50% in human hepatocyte proliferation, as revealed by the proliferation marker EdU in whole mount staining (Supplementary Fig. 5d–g). The effect of MYDGF could be intensified by a daily treatment of the human organoids with MYDGF for seven days. This resulted in a larger organoid area as well as a significant, two- to fourfold increase in hepatocyte proliferation (Fig. 4b–i). Thus, consistent with our data on human hepatocytes grown in 2D, MYDGF also had a growth-promoting effect on the more physiologic human organoids that were grown in 3D.

### MYDGF promotes hepatocyte proliferation after PHx in mice

**Fig. 5:**
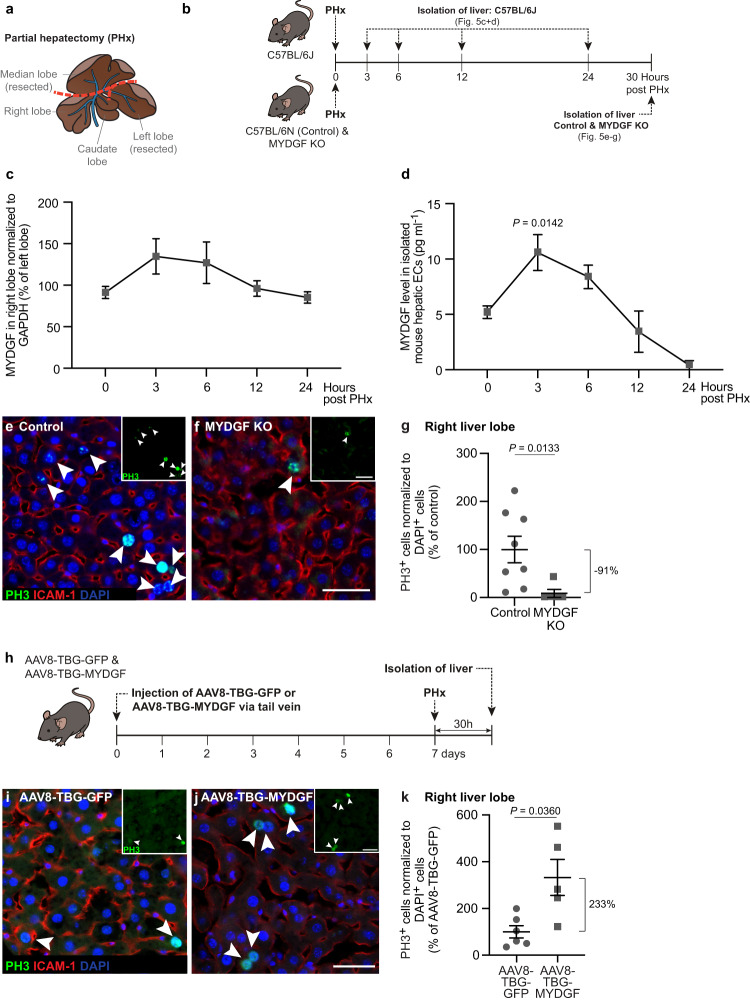
MYDGF stimulates proliferation of hepatocytes in the mouse liver after partial hepatectomy. **a** Anatomy of the mouse liver. **b** Schematic illustration of the experimental setup to determine myeloid-derived growth factor (MYDGF) expression kinetics and the effect of a global MYDGF knockout (KO) on hepatocyte proliferation after partial hepatectomy (PHx). **c** MYDGF protein levels in the right liver lobe relative to the respective left liver lobe of the same mouse, normalized to GAPDH protein levels; *n* = 5 (0 and 3 h), *n* = 4 (6 and 12 h) and *n* = 3 mice (24 h). **d** MYDGF protein levels in lysates of isolated mouse hepatic endothelial cells (ECs) from the right liver lobe; *n* = 5 (0 and 3 h), *n* = 4 (6 and 12 h) and *n* = 3 mice (24 h). Laser scanning microscopy (LSM) images of the right liver lobes of control (**e**) and MYDGF KO (**f**) mice. Phospho-Histone H3 (PH3, green, shown in inset), intercellular adhesion molecule-1 (ICAM-1, red), and DAPI (blue). **g** PH3^+^ (ICAM-1^−^) cells normalized to DAPI^+^ cells in control versus MYDGF KO mice, shown as percentage of control. *n* = 8 control versus *n* = 5 MYDGF KO transversal sections of the right liver lobe. **h** An adeno-associated-virus of serotype 8 (AAV8) driving under the control of a *thyroxine binding globulin* (TBG) promoter, green fluorescent protein (GFP) or MYDGF, was injected into the tail vein of mice. Seven days post injection, a PHx was performed, and the livers were isolated another 30 h later. **i**, **j** LSM images of right mouse liver lobes. PH3 (green, shown in inset), ICAM-1 (red) and DAPI (blue). **k** PH3^+^ (ICAM-1^−^) cells normalized to DAPI^+^ cells in AAV8-TBG-GFP versus AAV8-TBG-MYDGF mice, shown as percentage of AAV8-TBG-GFP. *n* = 6 AAV8-TBG-GFP versus *n* = 5 AAV8-TBG-MYDGF transversal sections of the right liver lobe. Scale bars: 50 µm (**f**, **j**). Data are presented as mean ± SEM. *P* values were calculated using one-way ANOVA followed by Dunnett’s post hoc test (**c**, **d**), and two-tailed unpaired Student’s *t*-test with Welch’s correction (**g**, **k**). Source data are provided as a Source Data file.

Based on our in vitro results, we hypothesized that MYDGF also had a growth-promoting effect on the mouse liver in vivo (Fig. 5). First, we examined MYDGF expression kinetics in mice (C57BL/6J) after performing a two-thirds PHx, in which the median and the left lobes of the liver were removed (Fig. 5a, b). After the PHx, MYDGF levels increased in the right liver lobe relative to the respective left liver lobe of the same mouse. Whereas MYDGF expression levels were elevated between 3 to 6 h after PHx, they returned to basal levels after 12 h (Fig. 5c). These data corresponded to the situation of hepatic endothelial cells that were isolated from the right liver lobe before and at different timepoints after PHx. Notably, 3 h after PHx, MYDGF expression levels peaked in hepatic ECs, stayed elevated until 6 h following the PHx, and then started to substantially decline (Fig. 5d).

Next, we performed in vivo loss-of-function experiments. More specifically, we used previously established *Mydgf*-deficient mice to determine whether MYDGF was required for hepatocyte proliferation after PHx^27^. Experimentally, we performed two-thirds PHx in control (C57BL/6N) and MYDGF KO mice (on C57BL/6N background), and we analyzed the right and caudate liver lobes 30 h after the surgery when hepatocyte proliferation normally increased after a PHx. Immunohistochemical analyses of the right and caudate liver lobes (stained for the proliferation marker PH3) showed that the proliferation rates in liver tissue from *Mydgf*-deficient mice were significantly lower after PHx in comparison to control mice (Fig. 5e–g, Supplementary Fig. 6a–c). An additional proliferation marker, that is Ki67, was used and co-stained with the hepatocyte marker HNF4α to analyze hepatocyte proliferation. This staining showed that in both liver lobes, hepatocytes barely proliferated in *Mydgf*-deficient mice compared to the respective liver lobes of control mice that underwent PHx (Supplementary Fig. 6d–i).

We then performed in vivo gain-of-function experiments and used an adeno-associated-virus of serotype 8 (AAV8) to drive *Mydgf* gene expression under the control of a liver-specific promoter, i.e*., thyroxine binding globulin* (TBG)^44,45^, to ensure that MYDGF is overexpressed in the liver, but not in other organs. Mice were injected with 1 × 10^12^ genome copies AAV8-TBG-green fluorescent protein (GFP) as the control group and AAV8-TBG-MYDGF via the tail vein. Seven days after infection of mice with the AAV8 viruses, a two-thirds PHx was performed. Thirty hours later, the remaining right and caudate liver lobes were isolated and prepared for analysis (Fig. 5h). First, we checked in the AAV8-TBG-GFP mice whether AAV8-TBG resulted in the expression of GFP in the liver (Supplementary Fig. 7a–c). Immunohistochemical staining of the right and caudate liver lobes showed a GFP signal, whereas GFP was not detectable in heart, kidney and spleen (Supplementary Fig. 7a). In the median lobe, we found that approximately 50% of the hepatocytes from AAV8-TBG-GFP mice expressed GFP to a variable extent (Supplementary Fig. 7b, c). In addition, we compared the MYDGF protein concentrations of the right liver lobe of AAV8-TBG-GFP mice with those of AAV8-TBG-MYDGF mice via Western blots (Supplementary Fig. 7d, e). The MYDGF protein levels were around 90% higher in the MYDGF group compared to the control group, thus showing a moderate (rather than excessive) overexpression of the MYDGF protein in the mouse liver. Immunohistochemical analyses of the right and caudate liver lobes stained for the proliferation marker PH3 and Ki67 showed that MYDGF was able to significantly enhance the proliferation rate in the mouse liver after PHx (Fig. 5i–k, Supplementary Fig. 8a–i). Notably, 4 days after PHx, the liver-to-body weight ratio was increased by 10% in AAV8-TBG-MYDGF compared to AAV8-TBG-GFP mice, while hepatocyte proliferation did no longer differ at this timepoint (Supplementary Fig. 8j–l).

These experiments show that MYDGF is required and sufficient for accelerating hepatocyte proliferation shortly after PHx in mice. Consistent with the transient proliferative effect of MYDGF on hepatocytes after PHx in vivo, we also observed a significantly enhanced phosphorylation of MAPK (T202/Y204) 24 h after PHx and, 6 h later, a numerically increased phosphorylation of STAT3 (S727) in the regenerating mouse liver following transfection with MYDGF (Supplementary Fig. 9). Together, our in vivo data on the regenerating liver provided further evidence for a growth-promoting effect of MYDGF.

## Discussion

Angiocrine signals, such as growth factors, cytokines, chemokines, extracellular matrix components and extracellular nucleotides have become a major focus of biomedical research over the last two decades^14^. Angiocrine signals guide differentiation, growth and polarization of the pancreas and the liver in the embryo^46–49^ as well as physiological and pathological processes in adult tissues, such as the pancreas, heart, lung, bone marrow, bone, brain, and liver^14,50–52^. The latter organ represents the most widely used tissue to study angiocrine signals in adult tissue regeneration^5,11–13,15–19,53–56^, since regeneration in the liver can be easily induced by partial hepatectomy (PHx). However, with the exception of HGF^24,57,58^, to our knowledge, no angiocrine signals have been identified yet to stimulate human or mouse hepatocyte proliferation on its own in vitro^20,59^.

Recently, we have shown that after PHx in mice, more blood flows through the remaining liver, so that its small blood vessels (i.e., liver sinusoids) dilate, resulting in mechanical stretching of hepatic ECs^5^. In addition, we showed that mechanical stretching of hepatic ECs in vitro triggers the release of angiocrine signals that stimulate proliferation and promote survival of in vitro cultivated primary human hepatocytes^5^. Therefore, we hypothesized that as yet unidentified, growth-promoting angiocrine signals are present in the supernatants of mechanically-stretched human hepatic ECs. Using a proteome-based screen, we surprisingly identified MYDGF as a secreted protein from mechanically-stretched hepatic ECs that has not been described previously as an angiocrine signal. Notably, the 3D molecular structure of MYDGF has only recently been elucidated and its β-sandwich topology has no similarity to any known cytokine or growth factor, and a receptor for MYDGF has also not been identified yet^27,60,61^. Here we showed that MYDGF significantly increased the proliferation rate of primary hepatocytes isolated from three different human donors. Because of the 3D cellular arrangement, organoid cell cultures have recently been established to more physiologically mimic the in vivo situation^41–43,62^. Therefore, we also tested MYDGF in organoids grown from human hepatocytes and showed that MYDGF was also able to increase the proliferation rate of primary human hepatocytes in these 3D human organoids. Although MYDGF has already been studied in the heart^27–30,37,63^, pancreas^64^, kidney^65^, bone^38^, and liver^39,40,66^, our data demonstrate that EC-derived MYDGF represents an angiocrine signal to promote growth of in vitro grown human hepatocytes. Consistently, blood concentrations were observed to transiently increase in patients after liver resection, and the expression of MYDGF similarly increased transiently in hepatic ECs from mice after PHx. It remains unclear though whether MYDGF is only released upon liver resection or whether it is generally released after surgical interventions and organ injuries. The finding that plasma levels of MYDGF were increased in patients after acute myocardial infarction compared to patients with stable coronary artery disease^27^, however, points to the possibility that MYDGF represents a general signal to induce regenerative responses, at least in liver and heart. To show that MYDGF indeed promotes hepatocyte proliferation in vivo, we used genetic loss- and gain-of-function experiments. These experiments revealed that MYDGF was required and sufficient to promote hepatocyte proliferation in the first stages of liver regeneration in mice.

Liver diseases are increasing in today’s society due to the sedentary lifestyle and metabolic comorbidities, especially obesity and diabetes mellitus^67^. In particular, nonalcoholic fatty liver disease (NAFLD) can progress to nonalcoholic steatohepatitis (NASH), liver cirrhosis and hepatocellular carcinoma (HCC)^68^. Therefore, there is an urgent need for more frequent and successful liver transplants. Recently, a liver perfusion machine was developed that is able to make human livers functional again that could not be used for transplantation due to their poor organ quality^25^. Thus, perfusion appears to increase the success of liver transplantation^25,69,70^, which fits to our hypothesis that perfusion and mechanical stimulation of ECs leads to the release of growth-promoting and survival-supporting angiocrine signals. The identification of MYDGF as an angiocrine signal offers the opportunity that MYDGF or even other mechanically-induced angiocrine signals could be used for organ preservation, regeneration, and growth when used in a liver perfusion machine. Recently, MYDGF was shown to alleviate NAFLD^40^, and in the future, MYDGF and other signals (including purinergic signals)^11,19^ may contribute and be used to improve regenerative and organ-based therapies (e.g., transplantation). Thus, identification of growth-promoting signals might help to grow human livers from smaller human liver pieces.

In conclusion, our results firstly demonstrate by using an unbiased proteomic screen on primary human hepatic endothelial cells that MYDGF is secreted from hepatic ECs upon mechanical stimulation, which does not exclude that it is also produced by other hepatic cell types, such as stellate cells. Secondly, MYDGF is an angiocrine signal that was found to promote human (and mouse) hepatocyte proliferation and survival. Thirdly, genetic loss- and gain-of-function experiments revealed that MYDGF is required and sufficient to promote hepatocyte proliferation in the mouse after PHx in vivo. Given that few growth factors have been identified yet to promote human hepatocyte growth, identification of MYDGF and, in the future, its receptor/s could open up new avenues for more successful transplantation and growth of human liver tissue.

## Methods

Our research complies with all relevant ethical regulations. Human study protocols were approved by the local institutional review board (Heinrich Heine University, Duesseldorf, Germany; study-no.: 2018–258-KFogU) and by the Landesärztekammer Rheinland-Pfalz (2020-15149). All participants were properly informed about the study and have signed in the informed consent form. The animal study protocol was approved by the German animal protection laws (Animal Ethics Committee of the Landesamt für Natur, Umwelt und Verbraucherschutz, Nordrhein-Westfalen).

### Mice

Male C57BL/6N mice (Charles River) and MYDGF KO mice at 10–15 weeks of age were used for two-thirds partial hepatectomy (PHx). MYDGF KO mice have recently been described^27^. The mice were generated on a BALB/c background and back-crossed to a C57BL/6N background (Fig. 5 and Supplementary Fig. 6). Male C57BL/6J mice (Janvier) at 10–15 weeks of age were used for adeno-associated virus serotype 8 (AAV8) injections and PHx (Fig. 5 and Supplementary Figs. 7–9). Mice were kept in rooms with controlled temperature at 22 °C, humidity of 55% and lighting from 6 a.m. to 6 p.m., and fed with the standard laboratory rodent chow (Ssniff, rat/mouse maintenance, V1534-300) and water ad libitum.

### Tail vein injections and two-thirds PHx

C57BL/6J mice were placed in a restrainer and the tail was immersed in warm water to dilate the blood vessels. 100 µl of AAV8-TBG-GFP or AAV8-TBG-MYDGF suspension (1 × 10^12^ genome copies) was slowly injected into the tail vein of the mice. Large scale AAV8-TBG-GFP and AAV8-TBG-MYDGF production and purification was performed by Vigene Biosciences, and the AAVs were also purchased from Vigene Biosciences. Seven days after injection a two-thirds PHx was performed according to the protocol from Mitchell and Willenbring^71^. PHx was also performed with C57BL/6N and MYDGF KO mice. Mice received 5 mg/kg Rimadyl (Carprofen, Zoetis) intraperitoneally as an analgesic and were anesthetized with 1.5–2% isoflurane. Then the median and left liver lobes were resected and afterwards the mice were kept warm to avoid hypothermia. After 3 h, 6 h, 12 h, 24 h, 30 h or 96 h the mice were sacrificed via cervical dislocation and the right and caudate liver lobes were isolated. Mice with improperly perfused (light brown) livers and hemorrhages in the livers were excluded from further analysis. For immunohistochemical analysis, the caudate liver lobe and half of the right liver lobe were placed in 4% paraformaldehyde (PFA, Thermo Fisher Scientific, J 19943). The other half of the right liver lobe was directly frozen in liquid nitrogen and stored at −80 °C for preparation of protein lysates. For magnetic activated cell sorting (MACS), 20 mg of the right liver lobe was removed for isolation of protein. The remaining right liver lobe was used for magnetic isolation of hepatic endothelial cells (ECs).

### Cryo-sections and immunohistochemical staining of liver lobes

The median liver lobe (resected during PHx) and the caudate and half of the right liver lobe (both remaining during PHx) of C57BL/6J, C57BL/6N and MYDGF KO mice, and AAV8-TBG-GFP and AAV8-TBG-MYDGF mice, and reference organs (heart, spleen, kidney) were equilibrated in 15% and 30% sucrose and placed transversally in Peel-A-Way® embedding molds (Polysciences, 18986-1) filled with Tissue-Tek® O.C.T^TM^ embedding medium (Sakura, 4583). 12-µm cryo-sections were prepared using a cryostat microtome HM 560 (Thermo Fisher Scientific). Sections were placed on Super-Frost slides (Thermo Fisher Scientific, J1800AMNZ) and stored at −20 °C until staining. For staining, slides were washed twice in phosphate buffered saline (PBS) containing Ca^2+^ and Mg^2+^ (PBS^+^) and once in PBS^+^ containing 0.2% Triton X-100 (AppliChem) for 5 min at RT. The slides were then blocked for 1 h at RT in PBS^+^ containing 0.2% Triton X-100 (AppliChem) and 1% bovine serum albumin (BSA, AppliChem), and the primary antibodies goat anti-ICAM-1/CD54 (R&D, AF796, 1/100), rabbit anti-phospho-Histone H3 (Ser10) (Sigma, 06-570, 1/50), rat anti-Ki-67 (SolA15) (Invitrogen, 14-5698-82, 1/100), rabbit anti-HNF4α (Abcam, ab181604, 1/100) and goat anti-GFP (Sicgen, AB0020-200, 1/500) were incubated at 4 °C. The next day, slides were washed twice in PBS^+^ and once in PBS^+^ containing 0.2% Triton X-100 for 10 min. Secondary antibodies were used as follows and incubated for 45 min at RT: donkey anti-goat/rabbit Alexa Fluor 555 (Invitrogen, A-21432/A-31572, 1/500), donkey anti-rabbit/rat/goat Alexa Fluor 488 (Invitrogen, A-21206/A-21208/A-11055, 1/500) and DAPI (Sigma-Aldrich, D9542, 1/1000). Before mounting with Fluoroshield^TM^ (Sigma-Aldrich, F6182), slides were washed thrice for 10 min in PBS^+^. To analyze proliferation, five images of up to four sections were acquired at 20x magnification using the laser scanning microscope (LSM 710, Zeiss). Proliferating cells were counted manually with Fiji (open-source software focused on biological-image analysis) or QuPath^72^, and DAPI^+^ cells were analyzed automatically by setting an individual threshold and applying it uniformly to each image using Fiji. All images were analyzed in a blinded fashion.

### In-situ split liver resection and liver resection

The surgical techniques used for in-situ split liver resection and liver resection have been described elsewhere^36,73^. MYDGF in human serum was determined in a male patient (70–80 years old) with a sigmoid carcinoma metastatic to the liver at different timepoints pre/post stage 1 in-situ split liver surgery (trisegmentectomy, i.e., segments 1 and 4–8). MYDGF level in human plasma after liver resection was analyzed in human individuals with hepatocellular carcinoma. Patients: Male, between 54–89 years old with a body mass index (BMI, kg m^−2^) of 22.8–28.7 and the following different resections: Laparoscopic left lateral resection (Seg 2/3); Hemihepatectomy right (Seg 5–8); Hemihepatectomy left (Seg 2–4); Open anterolateral sectorectomy (Seg 5/8); Open atypical resection segment 8; Open atypical resection (Seg 7/8). Individuals with obesity (BMI > 30 kg m^−2^) were excluded from the analysis, since a correlation between systolic blood pressure (that stretches hepatic ECs) and liver size could be demonstrated only in metabolically healthy individuals without obesity^5^. Blood serum and plasma of aforementioned patients were used for human MYDGF/SF20 ELISA (LSBio, LS-F13143), according to the manufacturer’s instructions. Protein levels were detected using the GloMax® Discover (Promega, Version 3.2.3).

### Human hepatic endothelial cells, human hepatocytes and HepG2 cells

Human hepatic endothelial cells (ECs) from different donors were purchased from PELOBiotech (PB-CH-153-5511). Donors: Female, 27-year-old, White, body mass index (BMI, kg m^−2^): 24.9, cause of death (COD): Anoxia (566.01.01.01.1 T); Male, 52-year-old, White, BMI: 30.6, COD: Anoxia (QC12B15F11); Female, 59-year-old, White, BMI: 18; COD: Anoxia second to Cardiovascular (QC29B15F09). Hepatic ECs were thawed and cultured according to the protocol provided by PELOBiotech. Microvascular endothelial cell growth medium supplemented with microvascular endothelial cell growth kit enhanced (PELOBiotech, PB-MH-100-4099) was used as medium. For final experiments 120,000–200,000 hepatic ECs in passage 2–4 were cultivated on stretch chambers (STREX, STB-CH-04). Stretch chambers were previously coated with Speed Coating Solution (PELOBiotech, PB-LU-000-0002-00). Human hepatic ECs on stretch chambers were mechanically stretched with an automated cell stretching system (STREX, STB-140-04) for 1.5 h (30 min, 20% static stretch plus 1 h, 20% cyclic stretch (30 cycles/min)).

Human hepatocytes from different donors were purchased from Thermo Fisher Scientific (HU4248, HU8296, HU8373, HU8300, HU8339-A, HU8284, HU8287) and KaLy-Cell (S1426T, B1148T). HU4248, HU8296, HU8339-A and HU8284 are no longer available from the company. Donors: Female, 12-year-old, White, BMI: 20.2, COD: Intracerebral hemorrhage-stroke (Lot # HU4248); Male, 23-year-old, White, BMI: 24.6, COD: Head trauma (Lot # HU8296); Female, 26-year-old, White, BMI: 18.6, COD: Asphyxiation (Lot # HU8373); Male, 31-year-old, White, BMI: 21, COD: Intracerebral hemorrhage (Lot # HU8300); Female, 31-year-old, African American, BMI: 18.9, COD: Asphyxiation (Lot # HU8339-A); Female, 46-year-old, White, BMI: 30.2, COD: Self-inflicted gunshot wound (Lot # HU8284); Female, 49-year-old, White, BMI: 19.6, COD: Head trauma (Lot # HU8287); Female, 34-year-old, White, BMI: 27.6, COD: Cholangiocarcinoma (Lot # S1426T); Female, 27-year-old, White, COD: Focal nodular hyperplasia, Budd-Chiari Syndrome (Lot # B1148T). Human hepatocytes were thawed and cultured according to the protocol provided by Thermo Fisher Scientific. For cultivation of hepatocytes in a 2D layer William’s E medium plus primary hepatocyte thawing/plating supplements and William’s E medium plus maintenance supplements (Gibco/Thermo Fisher Scientific, A1217601, CM3000, CM4000) were used. For growth and cultivation of hepatocyte organoids, we used a medium based on the protocol of Hu et al.^42^ and Garnier et al.^43^: Advanced DMEM/F-12 plus 1x Penicillin-Streptomycin-Glutamine, 10 mM HEPES, 1x B-27 Supplement (Gibco/Thermo Fisher Scientific, 12634010, 10378016, 15630106, 17504044), 500 ng/ml R-Spondin-1 (PeproTech, 120-38), 3 µM CHIR 99021 (Tocris, 4423), 1.25 mM N-Acetyl-L-cysteine (Sigma-Aldrich, A9165-5G), 10 mM Nicotinamide (Sigma-Aldrich, N0636-100G), 10 nM Gastrin I (Tocris, 3006), 50 ng/ml EGF (R&D Systems, 236-EG-200), 20 ng/ml TGF-α, 50 ng/ml FGF-7 (both PeproTech, 100-16 A and 100-19), 50 ng/ml FGF-10, 25 ng/ml HGF (both Miltenyi Biotec, 130-093-850 and 130-093-872), 2 µM A 83-01 (Tocris, 2939) and 10% Matrigel®Matrix (Corning, 356231). For the first three days of cultivation additionally 25 ng/ml Noggin (PeproTech, 120-10 C), 50 ng/ml Wnt-3a (R&D Systems, 5036-WN-010) and 10 µM Y-27632 (STEMCELL Technologies, 72302) were added. Organoids were grown and cultured in 24-well low attachment plates (Merck, CLS3473-24EA) in a humidified atmosphere at 5% CO_2_ and 37 °C.

HepG2 cells were purchased from ATCC (ATCC® HB-8065^TM^). The cells were thawed and cultured according to the protocol provided by ATCC in complete growth medium consisting of ATCC-formulated Eagle’s Minimum Essential Medium (ATCC, 30-2003) and 10% fetal bovine serum (FBS, Gibco, 10270-106).

### MYDGF treatment

For MYDGF treatment, 2D human hepatocytes were cultured on Permanox Chamber Slides (Thermo Fisher Scientific, C7182) or on 24-well plates (Sarstedt, 83.3922) pre-coated with 50 µg/ml rat tail collagen 1 (Thermo Fisher Scientific, A10483) diluted in speed coating solution (PELOBiotech, PB-LU-000-0002-00) o/n at RT, and human hepatocyte organoids on 24-well low attachment plates (Merck, CLS3473-24EA). 2D human hepatocytes with lot number HU4248, HU8300, HU8296, HU8373, HU8339-A, S1426T and B1148T were treated in a blinded manner with or without 1 ng/ml human MYDGF (Novoprotein, CG64) and hepatocytes with lot number HU8287 with or without 5 ng/ml MYDGF (Novoprotein, CG64) for 6 h. To identify the signaling pathway, hepatocytes (Lot # HU8373) were starved for 4 h in William’s E medium and incubated afterwards with or without 1 ng/ml MYDGF for 0, 5, 15 and 30 min, or hepatocytes were starved for 3 h in William’s E medium and pretreated with 20 µmol/l PD98059 (Sigma-Aldrich, P215) and/or 20 µmol/l Stattic (Sigma-Aldrich, S7947) for 1 h. After the pretreatment, cells were treated with or without 1 ng/ml MYDGF plus 20 µmol/l PD98059 and/or 20 µmol/l Stattic for 0, 5 and 15 min, and then used for the preparation of protein lysates. Hepatocytes (Lot # HU8373, HU8300, B1148T) were pretreated for 1 h with 20 µmol/l PD98059 and/or 20 µmol/l Stattic to analyze the number of proliferating hepatocytes after MYDGF treatment with or without inhibitors. After the pretreatment, 1 ng/ml MYDGF alone or 1 ng/ml MYDGF plus 20 µmol/l PD98059 and/or 20 µmol/l Stattic was added for 6 h. Hepatocyte organoids were treated with 10 ng/ml MYDGF (Novoprotein, CG64) once (Lot # HU8296) or daily (Lot # HU8296 and HU8373) after their formation, and they were cultured for 7 days, changing the medium every 2–3 days. For human hepatocyte proliferation, 2D hepatocytes were incubated simultaneously to MYDGF treatment with 1 mg/ml EdU (Thermo Fisher Scientific, C10337), and the 3D hepatocyte organoids were supplemented with 1 mg/ml EdU at every media exchange. HepG2 cells were cultured on 24-well plates (Sarstedt, 83.3922) and treated with 1 ng/ml MYDGF (Novoprotein, CG64) for 6 h.

### Galectin-3, CTGF and supernatant treatment

For Galectin-3 and CTGF treatment, 2D human hepatocytes were cultured on Permanox Chamber Slides (Thermo Fisher Scientific, C7182). Human hepatocytes with lot number HU8284 and HU8296 were treated with 6.25 µg/ml Galectin-3 (R&D, 1154-GA-050) or 1.5 µg/ml CTGF (Thermo Fisher Scientific, RP-8618) for 6 h. As an internal control and to demonstrate that human hepatocytes are able to respond with increased proliferation, hepatocytes were treated with the supernatant from unstretched and stretched hepatic ECs for 6 h.

### Mass spectrometry

Supernatants from unstretched and stretched hepatic ECs (3.5–3.6 ml) were centrifuged at 85,000 × *g*, 4 °C for 30 min and afterwards concentrated via centrifugal filters with a 3 kDa cut-off (Merck, Amicon Ultra UFC500324) following the manufacturer’s protocol. From each of the resulting concentrated supernatants 20 µl were loaded onto a short SDS-Page (10% polyacrylamide, 0.5 cm separation distance as previously described^74^). Gels were fixed in 30% methanol/7% acetic acid (v/v) (AppliChem) and stained with Coomassie blue (AppliChem) for visualization. Subsequently, protein bands were excised and subjected to in-gel protease digestion.

Gel slices were alternately washed four times with 25 mM ammonium bicarbonate (AppliChem) and 25 mM ammonium bicarbonate with 50% Acetonitrile (ACN, (v/v), Thermo Fisher Scientific). Protein reduction was performed in 65 mM dithiothreitol (DTT, AppliChem) for 15 min, shaking at 350 rpm and 50 °C. Subsequent alkylation was done in 216 mM iodoacetamide (AppliChem) for 15 min in the dark at room temperature. After washing (25 mM ammonium bicarbonate and 25 mM ammonium bicarbonate with 50% ACN (v/v)), gel slices were shrunk in 100% ACN. Protein digestion was performed with 400 ng LysC/Trypsin mix (Promega, V5073) in 25 mM ammonium bicarbonate and 2% ACN (v/v) over night at 37 °C. Resulting peptides were eluted with 1% trifluoroacetic acid (TFA, v/v), Thermo Fisher Scientific) followed by a second elution with 0.1% TFA/90% ACN (v/v). Peptides were lyophilized and subjected to MS analysis.

Lyophilized peptides were reconstituted in 1% TFA (v/v) supplemented with iRT peptides (iRT Kit, Biognosys, Ki-3002-1) and separated by liquid chromatography (LC, Ultimate 3000, Thermo Fisher Scientific) using an EASYspray ion source equipped with an Orbitrap Fusion Lumos mass spectrometer (Thermo Fisher Scientific). Peptides were trapped and desalted on an Acclaim PepMap 100 C18 LC trap column (Thermo Fisher Scientific, 164535) and subsequently separated via EASY-Spray C18 column (Thermo Fisher Scientific, P/N ES803) using a 100 min linear gradient from buffer A (0.1% formic acid) to 4–34% buffer B (80% ACN, 0.1% formic acid) at a flow rate of 300 nl/min followed by a 20 min linear gradient increasing buffer B to 50% and a 1 min linear gradient increasing buffer B to 90%. Column temperature was set to 40 °C.

For identification MS data were acquired in DDA (data dependent acquisition) mode. MS spectra were obtained at 120,000 resolution (3 s cycle time), m/z range of 350–1600 and a target value of 4e5 ions, with maximum injection time of 50 ms. For fragmentation precursor selection filter were set to charge state between 2 and 7, dynamic exclusion of 30 s and intensity threshold of 2.5e4. Fragmentation of precursors was done with an isolation window (m/z) 1.2, HCD energy of 32%, Orbitrap resolution of 15,000 and an ion target value of 1.0e5 with maximum injection time of 50 ms.

For identification MS spectra were analyzed with Proteome Discover (Thermo Fisher Scientific, Version 2.2.0.388). Used HTSequest search settings were: enzyme trypsin (full), max missed cleavages 2, peptide length 6-144, fragment mass tolerance 0.04 Da, modifications: carbamidomethyl (C) (fixed); oxidation (M), acetyl (protein N-term) (dynamic) and FASTA files (Homo sapiens (SwissProt TaxID 9606 (v2017-10-25)) Bos taurus (SwissProt TaxID 9913 and (v2017-10-25) and a general contaminant fasta file, consisting of ~250 sequences from different origin). For peptide spectrum match (PSM) validation Percolator node was used with standard settings (for input data max delta Cn 0.05, decoy database target FDR 0.01 (strict) and 0.05 (relaxed) and validation based on *q*-value). For protein grouping only rank 1 peptides were used and the parsimony principle were applied with protein FDR 0.01 (strict) and 0.05 (relaxed). For protein distribution across different samples, only unique peptides based on protein groups were used. Proteins that originate from contaminants, bovine, or with an undefined origin, either from bovine or from human were not considered for further analysis. The mass spectrometry proteomics data have been deposited to the ProteomeXchange Consortium via the PRIDE^75^ partner repository with the dataset identifier PXD033942.

### Flow cytometry in human hepatic ECs

Flow cytometry analysis was performed to determine the number of dead cells from unstretched and stretched hepatic ECs. The medium (possibly containing detached dead cells) was transferred to a tube (Falcon, 352052) and adherent ECs were subsequently detached by trypsinization and transferred to the corresponding tube. Tubes were centrifuged (400 × *g*, 5 min) and cells were washed with PBS (Gibco, 10010-015). Centrifugation step was repeated, and FVS660 (BD Biosciences, 564405, 1/1000) diluted in 1x PBS was added for 15 min at RT in the dark. Cells were washed with PBS and centrifuged for 3 min at 400 × *g*. Cell pellet was resuspended in PBS, and FVS660 positive (FVS660^+^, dead cells) and FVS660 negative (FVS660^-^, living cells) cells were determined using CytoFlex S (Beckman Coulter, CytExpert Version 2.4.0.28). For quantification FlowJo software version 10 (BD Biosciences, RRID:SCR_008520) was used.

### Western blotting

First, Western blotting was performed to analyze MYDGF levels in supernatants and GAPDH levels in lysates of unstretched and stretched hepatic ECs. The supernatant of unstretched and stretched hepatic ECs was concentrated with Amicon Ultra-0.5 centrifugal filter units (Merck, UFC501096). 500 µl of supernatant were centrifuged twice for 15 min at 15,700 × *g* and 4 °C. Second, lysates of unstretched and stretched hepatic ECs, lysates of human hepatocytes (Lot # HU8373) and lysates of the right and left liver lobes of mice were prepared. Lysis buffer: 50 mM HEPES (Carl Roth), 150 mM NaCl (Carl Roth), 10% Glycerol (Carl Roth), 1% Triton X-100 (AppliChem), PhosSTOP^TM^ phosphatase inhibitor (Sigma-Aldrich, 4906845001) and cOmplete^TM^ protease inhibitor cocktail (Roche, 11697498001). Pierce^TM^ BCA^TM^ Protein Assay (Thermo Fisher Scientific, 23225) was used to determine the protein concentration of the cell and mouse lysates. Supernatants and lysates were heated in 2x Laemmli sample buffer (Bio-Rad, 1610747) containing β-mercaptoethanol (Carl Roth) at 95 °C for 5 min. For protein separation and Western blotting, the Bio-Rad system with Mini-Protean^TM^ TGX Stain-Free Protein Gels (Bio-Rad, 4568086 and 4568085) and the Trans-Blot® Turbo^TM^ Transfer System (Bio-Rad, 1704156) or Trans-Blot® Turbo^TM^ RTA Mini LF PVDF Transfer Kit (Bio-Rad, 1704274) was used. Rabbit anti-MYDGF (Proteintech, 11353-1-AP, 1/1000), rabbit anti-Phospho-p44/42 MAPK (Erk1/2) (Thr202/Tyr204) (Cell Signaling, 4376, 1/750 or 1/500), rabbit anti-p44/42 MAPK (Erk1/2) (137F5) (Cell Signaling, 4695, 1/750 or 1/500), rabbit anti-Phospho-Stat3 (Ser727) (Cell Signaling, 9134, 1/500), rabbit anti-Stat3 (D3Z2G) (Cell Signaling, 12640, 1/750), rabbit anti-Phospho-AKT (Ser473) (D9E) (Cell Signaling, 4060, 1/750), rabbit anti-AKT (pan) (C67E7) (Cell Signaling, 4691, 1/750) as well as rabbit anti-GAPDH (Abcam, ab9485, 1/5000 or 1/2000) and rabbit anti-β tubulin antibody (Abcam, ab6046, 1/2000) as loading controls were used as primary antibodies and incubated o/n at 4 °C. Goat anti rabbit IgG, HRP-linked (Cell Signaling, 7074, 1/2000) was used as secondary antibody and incubated for 45–60 min at RT. Chemiluminescence was detected with Clarity^TM^ Western ECL Substrate (Bio-Rad, 170-5061) or WesternBright^TM^ Peroxide Chemiluminescent Detection Reagent (Advansta, R-03025-D10) and visualized with ChemiDoc MP Imaging System (Bio-Rad, Image Lab Touch Software Version 2.4.0.03). Densitometrical analysis was performed using Image Laboratory Software Versions 5.2 and 6.1 (Bio-Rad).

### Magnetic activated cell sorting (MACS)

To isolate hepatic ECs from the remaining right liver lobe post PHx, a MACS method was used. Isolation was performed according to the protocols Liver Dissociation Kit mouse (Miltenyi Biotec, 130105807) and CD146 (LSEC) MicroBeads (Miltenyi Biotec, 130092007) provided by the company. Only the application of the cell suspension on the MS columns (Miltenyi Biotec, 130042201) was changed (to increase the purity of the isolated cells) as follows. A liver cell suspension was prepared, using the liver dissociation kit (Miltenyi Biotec, 130105807) and the gentleMACS^TM^ Octo Dissociator with heaters (Miltenyi Biotec, 130096427). Afterwards, mouse hepatic ECs were labeled with CD146+ magnetic beads (Miltenyi Biotec, 130092007), and the cell suspension was applied twice on two different MS columns, which were placed in a QuadroMACS^TM^ separator (Miltenyi Biotec, 130090976) on a MACS® MultiStand (Miltenyi Biotec, 130042303). To isolate the magnetically-labeled cells, which remained in the columns, the latter were removed from the magnetic field and flushed with PEB buffer (MACS® BSA stock solution (Miltenyi Biotec, 130091376) diluted 1/20 in autoMACS® rinsing solution (Miltenyi Biotec, 130091222). Cells were centrifuged for 5 min at 9000 × *g* and resuspended in 500 µl protein lysis buffer containing 50 mM HEPES (Carl Roth), 150 mM NaCl (Carl Roth), 10% Glycerol (Carl Roth), 1% Triton X-100 (AppliChem), PhosSTOP^TM^ phosphatase inhibitor (Sigma-Aldrich, 4906845001) and cOmplete^TM^ protease inhibitor cocktail (Roche, 11697498001). Lysates were stored at -80 °C, before mouse MYDGF ELISA (AVIVA Systems Biology, OKEI00375) was performed according to the manufacturer’s instructions. Protein levels were detected using the GloMax® Discover (Promega, Version 3.2.3).

### Quantitative real-time PCR

RNA from unstretched and stretched hepatic ECs, human hepatocytes and HepG2 cells was isolated using the High Pure RNA Isolation Kit (Roche, 11828665001) and cDNA was synthesized using the Super-Script^TM^ II Reverse Transcriptase (Thermo Fisher Scientific, 18064014) according to the protocols provided by the companies. The following primer sequences (purchased from Eurogentec) were used: Human *MYDGF* forward 5′–TCG TGC ATT CCT TCT CCC AT–3′ and human *MYDGF* reverse 5′–ACC TCT GCC TTG AAC TGT GT–3′; human *AFP* forward: 5´–ACA TCC TCA GCT TGC TGT CT–3′ and human *AFP* reverse 5´–AAT GCT TGG CTC TCC TGG AT–3′; human *RPLP0* forward 5′–GAA GAC AGG GCG ACC TGG AA–3′ and human *RPLP0* reverse 5′–CCA CAT TGT CTG CTC CCA CA–3′; Human *B2M* forward 5′–TTT CAT CCA TCC GAC ATT GA–3′ and human *B2M* reverse 5′–CCT CCA TGA TGC TGC TTA CA–3′; Human *HPRT* forward 5′–GCA GAC TTT GCT TTC CTT GG–3′ and human *HPRT* reverse 5′–AAC ACT TCG TGG GGT CCT TT–3′. Quantitative real-time PCR was run in duplicates by using the LightCycler Nano instrument (Roche) with the LightCycler® Nano Software 1.1 (Roche) or the QuantStudio^TM^ 1 Real-Time PCR Instrument (Applied Biosystems, A40425) with the QuantStudio^TM^ Design & Analysis Software v1.5.1 (Thermo Fisher Scientific). Relative gene expression of *MYDGF* or *AFP* was normalized to housekeeping genes and calculated by using the comparative C_T_ method^76^.

### Proliferation and cell death staining of 2D human hepatocytes and HepG2 cells

2D human hepatocytes on Permanox Chamber Slides (Thermo Fisher Scientific, C7182) were used for analysis of proliferation via EdU Click-iT® reaction (Thermo Fisher Scientific, C10214 and C10337) or phospho-Histone H3 (PH3) staining and apoptosis via cell death assay (in situ cell death detection kit (TUNEL), TMR red (Roche, 12156792910) or caspase-3 staining. For HepG2 cells the PH3 staining was performed to detect proliferating cells. Hepatocytes and HepG2 cells were fixed in 4% PFA (Thermo Fisher Scientific, J19943) o/n at 4 °C under agitation. EdU proliferation assay and cell death assay were performed according to the manufacturer’s instructions (Thermo Fisher Scientific, C10214 and C10337 and Roche, 12156792910). Protocols were followed by co-staining with rabbit anti-HNF4α (Cell Signaling, C11F12, 3113, 1/500) and/or DAPI (Sigma-Aldrich, D9542, 1/1000). For this and for PH3 and caspase-3 staining, slides were washed thrice for 5 min with PBS containing Ca^2+^ and Mg^2+^ (PBS^+^) and blocked in PBS^+^ supplemented with 0.3% Triton X-100 (AppliChem) and 5% normal donkey serum (NDS, Jackson ImmunoResearch) for 1 h at RT. HNF4α and rabbit anti-phospho-Histone H3 (Ser10) (Sigma-Aldrich, 06-570, 1/50) or rabbit anti-caspase-3 (Sigma-Aldrich, C8487, 1/200) were diluted in antibody dilution solution containing 0.3% Triton X-100 (AppliChem) and 1% bovine serum albumin (BSA, AppliChem) and incubated for 1 h at RT. Afterwards, slides were washed thrice for 5 min in PBS^+^ and incubated with donkey anti-rabbit Alexa Fluor 555 (Invitrogen, A-31572, 1/500) and donkey anti-rabbit Alexa Fluor 488 (Invitrogen, A-21206, 1/500) and DAPI (Sigma-Aldrich, D9542, 1/1000) for 1.5 h at RT. Slides were then washed thrice for 5 min in PBS^+^ and mounted with Fluoroshield^TM^ (Sigma-Aldrich, F6182). For the cell death assay, only co-staining with DAPI was performed. Slides were blocked in PBS^+^ containing 0.2% Triton X-100, 3% BSA and 5% NDS, and DAPI was incubated for 45 min. To analyze proliferation of human hepatocytes a tile scan image consisting of 7×7 tiles were acquired at 10x magnification using the laser scanning microscope (LSM 710, Zeiss). To analyze proliferation of HepG2 cells and apoptotic hepatocytes, five images per well were acquired at 20x magnification using the LSM (LSM 710, Zeiss). Proliferating cells were counted manually in Fiji in a blinded manner. TUNEL^+^ cells and caspase-3^+^ area were determined using a threshold that was chosen to be the same for each image. For quantification, PH3^+^ HepG2 cells were divided by DAPI^+^ cells and the TUNEL^+^ and caspase-3^+^ area was divided by the DAPI^+^ area.

### Proliferation and whole mount staining of 3D human hepatocyte organoids

Human hepatocyte organoids were transferred to 96-well µ-clear bottom plates (Greiner Bio-One, 655090) for EdU proliferation assay (Thermo Fisher Scientific, C10214 and C10337). Hepatocyte organoids were fixed in 4% PFA (Thermo Fisher Scientific, J19943) o/n at 4 °C. On the next day, organoids were washed thrice with PBS^+^ containing 0.1% Triton X-100 (wash buffer, AppliChem) and blocked for 30 min at RT in PBS^+^ containing 0.1% Triton X-100, 10% NDS and 1% BSA (blocking solution). The EdU proliferation assay was performed according to the manufacturer’s instructions (Thermo Fisher Scientific, C10214 and C10337). For (additional) whole mount staining, organoids were washed thrice with wash buffer and incubated with rabbit anti-HNF4α (Cell Signaling, C11F12, 3113, 1/250) diluted in blocking solution for 2 h at RT or o/n at 4 °C. Both before and after the addition of the secondary antibody, the organoids were washed thrice in wash buffer. The antibodies were as follows: donkey anti-rabbit Alexa Fluor 555 (Invitrogen, A-31572, 1/500) and DAPI (Sigma-Aldrich, D9542, 1/1000). The incubation time was 2 h at RT. All steps were performed under agitation. (Z-stack) images were acquired using LSM (LSM 710, Zeiss), and analyses were done with Fiji. Organoid area was measured with a free hand selection tool, and EdU^+^ cells were counted manually in a blinded manner.

### Cell viability assay of 3D human hepatocyte organoids

To show that human hepatocyte 3D organoids consist of viable cells, a Live-Dead Viability/Cytotoxicity Assay was performed (Thermo Fisher Scientific, L3224). Human hepatocyte organoids were transferred to a 96-well µ-clear bottom plate and stained for calcein (2 µm, green), ethidium homodimer-1 (EthD-1, 4 µm, red) and Hoechst (10 µg/ml, blue, Thermo Fisher Scientific, H3570). Organoids were incubated with the dyes for 30 min at 37 °C and 5% CO_2_. Z-stack images were acquired using the laser scanning microscope (LSM 710, Zeiss) and representative maximum intensity projections were processed with Fiji.

### Statistics and reproducibility

Except for the paired data, all data are presented as mean ± SEM. *P* values were calculated using two-tailed paired or unpaired Student’s *t*-test with Welch’s correction, multiple unpaired *t*-test with Welch’s correction, one-way ANOVA with Dunnett’s post-hoc test and two-way ANOVA with Tukey’s or Šidák´s post-hoc test. Statistical significance was calculated using Excel (Microsoft) or Prism 10 (GraphPad). Outliers were excluded after performing a Grubbs’ test for outliers (*P* = 0.05) using Prism for Figs. 1e, f, 2e, f, l, 3b, d, f, 4i, Supplementary Figs. 3j and 4b, d, i. The following experiments were replicated: Stretch experiments with human hepatic ECs (shown in Fig. 1b–d), EdU proliferation assay (shown in Fig. 2a–f and Supplementary Fig. 2a–c) and PH3 staining (shown in Fig. 2g–i and Supplementary Fig. 2d–f) in 2D cultured human hepatocytes. TUNEL staining (shown in Fig. 2j–l and Supplementary Fig. 2k–n) and caspase-3 staining (shown in Fig. 2m–o and Supplementary Fig. 2o–q) in 2D cultured human hepatocytes. EdU proliferation assay in 3D cultured human hepatocytes (shown in Fig. 4b–i and Supplementary Fig. 5d–f). PH3 staining in control versus MYDGF KO mice (shown in Fig. 5e–g and Supplementary Fig. 6a–c) was reproduced with an additional proliferation marker, i.e., Ki67 (shown in Supplementary Fig. 6d–i). PH3 staining in AAV8-TBG-GFP versus AAV8-TBG-MYDGF mice (shown in Fig. 5i–k and Supplementary Fig. 8a–c) was reproduced with an additional proliferation marker, i.e., Ki67 (shown in Supplementary Fig. 8d–i).

### Reporting summary

Further information on research design is available in the Nature Portfolio Reporting Summary linked to this article.

### Supplementary information

Supplementary Information

Reporting Summary

### Source data

Source Data

## Data Availability

The mass spectrometry proteomics data have been deposited to the ProteomeXchange Consortium via the PRIDE^75^ partner repository with the dataset identifier PXD033942. All additional data is available upon request to the corresponding author (Eckhard Lammert, lammert@hhu.de). Source data are provided with this paper.
