# Supplementary material for: Identification of myeloid-derived growth factor as a mechanically-induced, growth-promoting angiocrine signal for human hepatocytes
Source: Nat Commun. 2024 Feb 5;15:1076. doi: 10.1038/s41467-024-44760-y (PMC10844291; doi:10.1038/s41467-024-44760-y)
Supplement: Supplementary file 1 — Supplementary Information [file 41467_2024_44760_MOESM1_ESM.pdf]

Identification of myeloid-derived growth factor as a mechanically-induced, growth-promoting angiocrine signal for human hepatocytes

Supplementary Table 1 (related to Figure 1): Proteins in the supernatants of unstretched and mechanically-stretched human hepatic endothelial cells identified by liquid chromatography tandem-mass spectrometry. Primary human hepatic endothelial cells (ECs) were mechanically-stretched, and the supernatants of unstretched and stretched hepatic ECs were analyzed using liquid chromatography tandem-mass spectrometry (LC-MS/MS). A total of 70 proteins were identified in the supernatants of unstretched and stretched hepatic ECs. 45 proteins were identified in the supernatants of both unstretched and stretched hepatic ECs (light grey). 22 proteins were identified exclusively in the supernatants of stretched hepatic ECs (white), and three proteins were identified exclusively in the supernatants of unstretched hepatic ECs (dark grey). None of these proteins were identified in the cultivation medium without the hepatic ECs.

UniProt Accession	Description	HGNC Symbol	Identified in Sample Group: Unstretched	Identified in Sample Group: Stretched
Q96FW1	Ubiquitin thioesterase otub1 [OS=Homo sapiens]	OTUB1		x
O00299	chloride intracellular channel protein 1 [OS=Homo sapiens]	CLIC1		x
P30086	phosphatidylethanolamine-binding protein 1 [OS=Homo sapiens]	PEBP1		x
P51688	N-sulphoglucosamine sulphohydrolase [OS=Homo sapiens]	SGSH		x
P07195	L-lactate dehydrogenase B chain [OS=Homo sapiens]	LDHB		x
Q969H8	Myeloid-derived growth factor [OS=Homo sapiens]	C19orf10; MYDGF		x
P67936	Tropomyosin alpha-4 chain [OS=Homo sapiens]	TPM4		x
P17931	Galectin-3 [OS=Homo sapiens]	LGALS3		x
P07686	Beta-hexosaminidase subunit beta [OS=Homo sapiens]	HEXB		x
Q9P109	beta-1,3-galactosyl-O-glycosyl-glycoprotein beta-1,6-N-acetylglucosaminyltransferase 4 [OS=Homo sapiens]	GCNT4		x
P35237	serpin B6 [OS=Homo sapiens]	SERPINB6		x
P43490	nicotinamide phosphoribosyltransferase [OS=Homo sapiens]	NAMPT		x
Q14019	coactosin-like protein [OS=Homo sapiens]	COTL1		x
P61916	Epididymal secretory protein E1 [OS=Homo sapiens]	NPC2		x
P41250	Glycine--tRNA ligase [OS=Homo sapiens]	GARS		x
Q15293	Reticulocalbin-1 [OS=Homo sapiens]	RCN1		x
O15144	Actin-related protein 2/3 complex subunit 2 [OS=Homo sapiens]	ARPC2		x
P15144	aminopeptidase N [OS=Homo sapiens]	ANPEP		x
P21980	Protein-glutamine gamma-glutamyltransferase 2 [OS=Homo sapiens]	TGM2		x
Q66K41-1	Zinc finger protein 385C [OS=Homo sapiens]	ZNF385C		x
P00441	Superoxide dismutase [Cu-Zn] [OS=Homo sapiens]	SOD1		x
Q92820	Gamma-glutamyl hydrolase [OS=Homo sapiens]	GGH		x
P02452	Collagen alpha-1(I) chain [OS=Homo sapiens]	COL1A1	x	
O76061	Stanniocalcin-2 [OS=Homo sapiens]	STC2	x	
P08572	Collagen alpha-2(IV) chain [OS=Homo sapiens]	COL4A2	x	
P01034	Cystatin-C [OS=Homo sapiens]	CST3	x	x
P00491	purine nucleoside phosphorylase [OS=Homo sapiens]	PNP	x	x
P31949	protein S100-A11 [OS=Homo sapiens]	S100A11	x	x
P50453	Serpin B9 [OS=Homo sapiens]	SERPINB9	x	x
P09211	Glutathione S-transferase P [OS=Homo sapiens]	GSTP1	x	x
Q9Y4K0	Lysyl oxidase homolog 2 [OS=Homo sapiens]	LOXL2	x	x
P37837	Transaldolase [OS=Homo sapiens]	TALDO1	x	x
P09110-1	3-ketoacyl-CoA thiolase, peroxisomal [OS=Homo sapiens]	ACAA1	x	x
Q9UBP4	Dickkopf-related protein 3 [OS=Homo sapiens]	DKK3	x	x
P52565	rho GDP-dissociation inhibitor 1 [OS=Homo sapiens]	ARHGDIA	x	x
P49327	Fatty acid synthase [OS=Homo sapiens]	FASN	x	x
P50454	Serpin H1 [OS=Homo sapiens]	SERPINH1	x	x
Q16658	Fascin [OS=Homo sapiens]	FSCN1	x	x
P08758	annexin A5 [OS=Homo sapiens]	ANXA5	x	x
P33151	Cadherin-5 [OS=Homo sapiens]	CDH5	x	x
Q9NPG4	Protocadherin-12 [OS=Homo sapiens]	PCDH12	x	x
P14174	Macrophage Migration inhibitory factor [OS=Homo sapiens]	MIF	x	x
P18669	Phosphoglycerate mutase 1 [OS=Homo sapiens]	PGAM1; LOC643576	x	x
P07996	thrombospondin-1 [OS=Homo sapiens]	THBS1	x	x
P46940	Ras GTPase-activating-like protein IQGAP1 [OS=Homo sapiens]	IQGAP1	x	x
P23284	peptidyl-prolyl cis-trans isomerase B [OS=Homo sapiens]	PPIB	x	x
P03956	Interstitial collagenase [OS=Homo sapiens]	MMP1	x	x
Q13085-4	Isoform 4 of Acetyl-CoA carboxylase 1 [OS=Homo sapiens]	ACACA	x	x
Q86VQ0	Lebercilin [OS=Homo sapiens]	LCA5	x	x
P61769	Beta-2-microglobulin [OS=Homo sapiens]	B2M	x	x
A6NI15	Mesogenin-1 [OS=Homo sapiens]	MSGN1	x	x
Q15113	Procollagen C-endopeptidase enhancer 1 [OS=Homo sapiens]	PCOLCE	x	x
P40926	Malate dehydrogenase, mitochondrial [OS=Homo sapiens]	MDH2	x	x
P13489	Ribonuclease inhibitor [OS=Homo sapiens]	RNH1	x	x
P11047	Laminin subunit gamma-1 [OS=Homo sapiens]	LAMC1	x	x
P29279-1	Connective tissue growth factor [OS=Homo sapiens]	CTGF	x	x
P26022	Pentraxin-related protein PTX3 [OS=Homo sapiens]	PTX3	x	x
P52566	Rho GDP-dissociation inhibitor 2 [OS=Homo sapiens]	ARHGDIB	x	x
P07942	Laminin subunit beta-1 [OS=Homo sapiens]	LAMB1	x	x
Q13201	Multimerin-1 [OS=Homo sapiens]	MMRN1	x	x
Q9BX63-2	Isoform 2 of Fanconi anemia group J protein [OS=Homo sapiens]	BRIP1	x	x
P09936	Ubiquitin carboxyl-terminal hydrolase isozyme L1 [OS=Homo sapiens]	UCHL1	x	x
P30101	Protein disulfide-isomerase A3 [OS=Homo sapiens]	PDIA3	x	x
P08123	Collagen alpha-2(I) chain [OS=Homo sapiens]	COL1A2	x	x
P04275	Von Willebrand factor [OS=Homo sapiens]	VWF	x	x
P35579-1	Myosin-9 [OS=Homo sapiens]	MYH9	x	x
P06703	protein S100-A6 [OS=Homo sapiens]	S100A6	x	x
P07858	Cathepsin B [OS=Homo sapiens]	CTSB	x	x
Q15582	Transforming growth factor-beta-induced protein ig-h3 [OS=Homo sapiens]	TGFBI	x	x
Q9UNN8	Endothelial protein C receptor [OS=Homo sapiens]	PROCR	x	x

a Unstretched

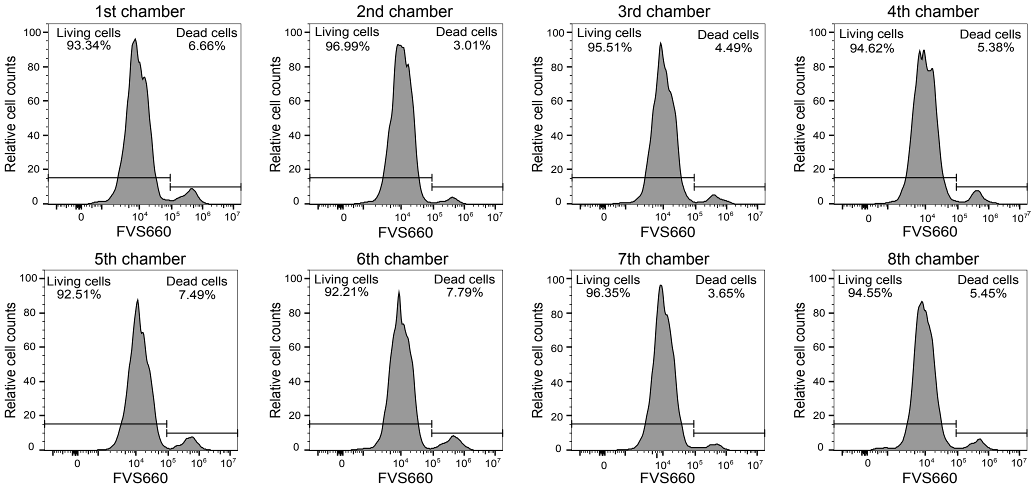

b Stretched

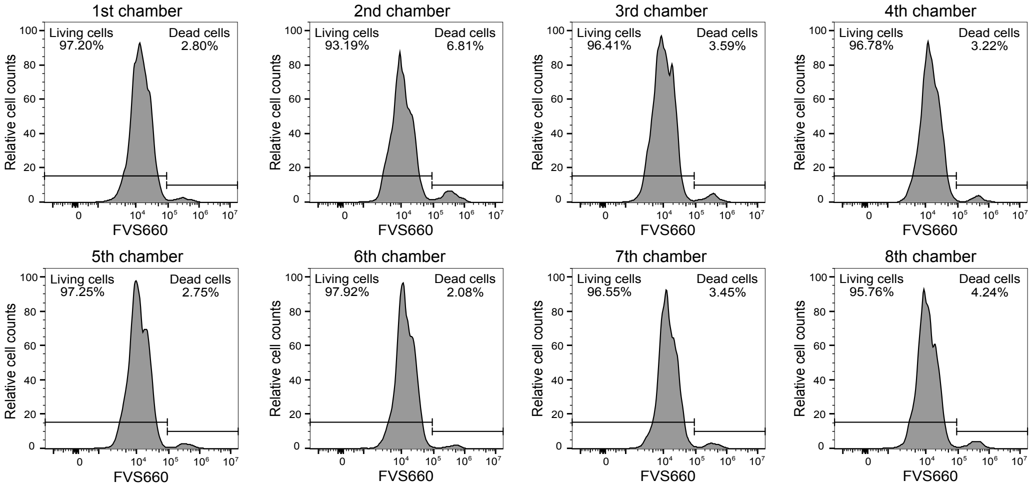

c Quantification

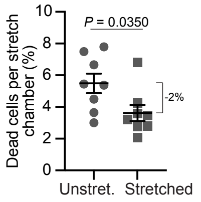

Supplementary Figure 1 (related to Figure 1): Viability of unstretched and mechanically-stretched human hepatic endothelial cells. a, b Flow cytometric analysis of cell viability using Fixable Viability Stain 660 (FVS660). Histograms show human hepatic endothelial cells (ECs) that were either unstretched (a) or mechanically-stretched (b). **c** Quantification of dead cells per stretch chamber with n = 8 chambers of unstretched (a) and mechanically-stretched (b) human hepatic ECs. Data in (c) are shown as percent of total number of cells and presented as mean ± SEM. *P* values were calculated using two-tailed unpaired Student's *t*-test with Welch's correction. Source data are provided as a Source Data file.

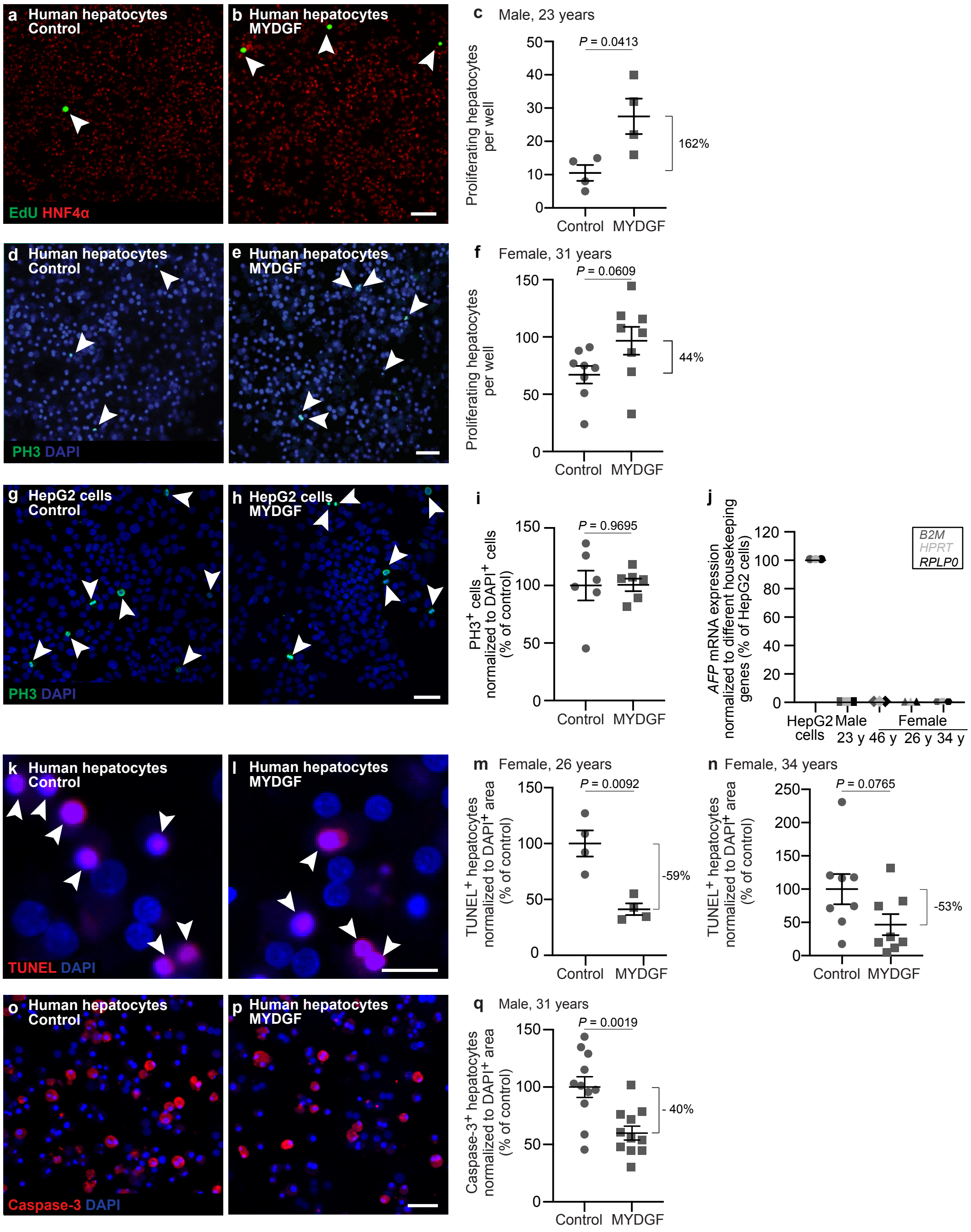

a Human hepatocytes Control
b Human hepatocytes MYDGF
EdU HNF4α
c Male, 23 years
P = 0.0413
Proliferating hepatocytes per well
162%
Control MYDGF
d Human hepatocytes Control
e Human hepatocytes MYDGF
PH3 DAPI
f Female, 31 years
P = 0.0609
44%
g HepG2 cells Control
h HepG2 cells MYDGF
i
P = 0.9695
PH3+ cells normalized to DAPI+ cells (% of control)
j
AFP mRNA expression normalized to different housekeeping genes (% of HepG2 cells)
B2M
HPRT
RPLP0
HepG2 cells
Male
Female
23 y 46 y 26 y 34 y
k Human hepatocytes Control
l Human hepatocytes MYDGF
TUNEL DAPI
m Female, 26 years
P = 0.0092
TUNEL+ hepatocytes normalized to DAPI+ area (% of control)
-59%
n Female, 34 years
P = 0.0765
-53%
o Human hepatocytes Control
p Human hepatocytes MYDGF
Caspase-3 DAPI
q Male, 31 years
P = 0.0019
Caspase-3+ hepatocytes normalized to DAPI+ area (% of control)
- 40%

Supplementary Figure 2 (related to Figure 2): MYDGF stimulates proliferation of primary human hepatocytes rather than liver cancer cell line HepG2. a, b Representative laser scanning microscopy (LSM) images of human hepatocytes treated without (a) or with (b) myeloid-derived growth factor (MYDGF). Arrowheads point to proliferating cells stained for 5-ethynyl-2'-deoxyuridine (EdU, green) and hepatocytes stained for hepatocyte nuclear factor 4α (HNF4α, red). **c** Quantification of proliferating hepatocytes in a male, 23-year-old donor, n = 4 wells of human hepatocytes. **d, e** Representative LSM images of human hepatocytes treated without (d) or with (e) MYDGF, stained for the proliferation marker phospho-Histone H3 (PH3, green), and DAPI (blue). **f** Quantification of proliferating human hepatocytes in a female, 31-year-old donor; n = 8 wells of human hepatocytes. **g, h** Representative LSM images of HepG2 cells treated without (g) or with (h) MYDGF, stained for PH3 (green) and DAPI (blue). **i** Quantification of $PH3^{+}$ HepG2 cells; n = 6 wells. **j** Quantification of *alpha-fetoprotein* (*AFP)* expression levels normalized to different housekeeping genes: *B2M, HPRT* and *RPLP0* in lysates of HepG2 cells and human hepatocytes from four different donors (male, 23-year-old and female 46-, 26- and 34-year-old); n = 1 cell lysate. **k, l** Representative LSM images of human hepatocytes treated without (k) or with (l) MYDGF. Apoptotic cells were visualized by TUNEL staining (red), and cell nuclei were counterstained for DAPI (blue). **m, n** Quantification of $TUNEL^{+}$ human hepatocytes. Donors: (m) female, 26 years, n = 4 wells each; (n) female, 34 years, n = 8 wells each. **o, p** Representative LSM images of human hepatocytes treated without (o) or with (p) MYDGF. Apoptotic cells were visualized by caspase-3 staining (red), and cell nuclei were counterstained for DAPI (blue). **q** Quantification of caspase-3^{+} human hepatocytes from a male, 31-year-old donor, n = 11 wells each. Scale bars: 100 μm (b), 50 μm (e, h, p) and 20 μm (l). Data are presented as mean ± SEM. *P* values were calculated using two-tailed unpaired Student's *t*-test with Welch's correction (c, f, i, m, n, q). Source data are provided as a Source Data file.

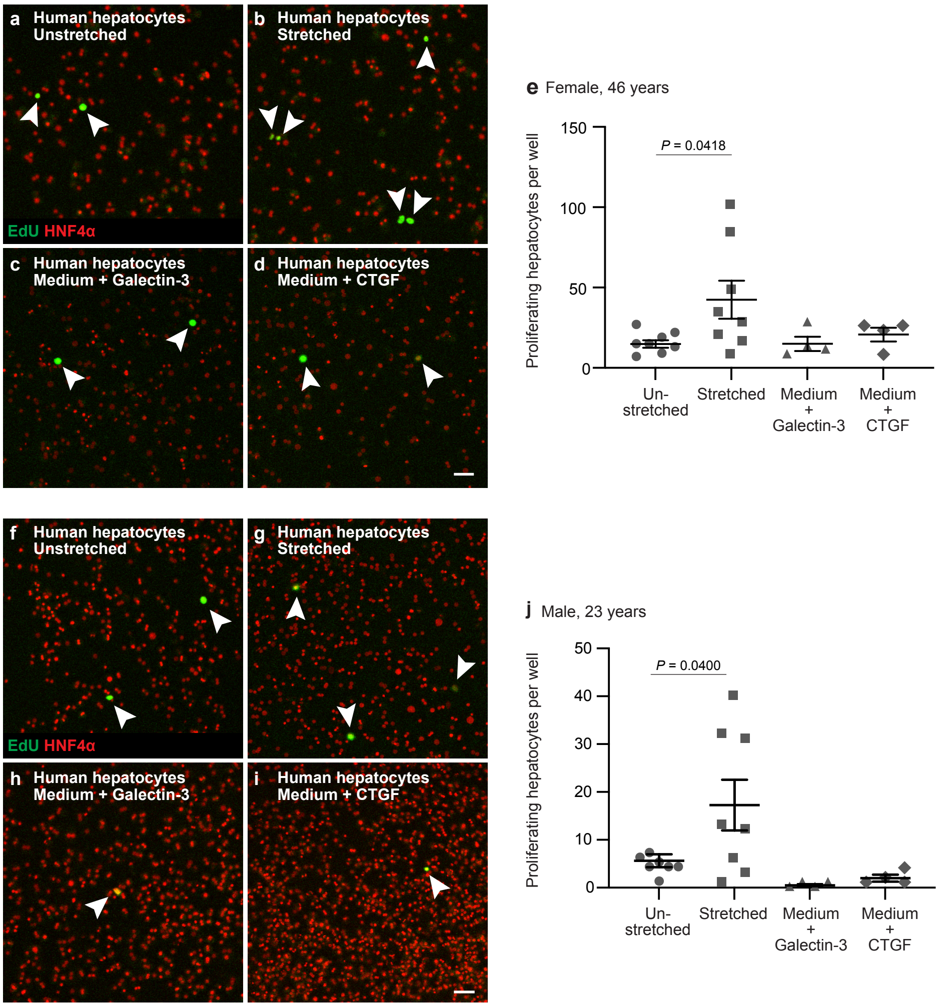
a Human hepatocytes Unstretched
b Human hepatocytes Stretched
EdU HNF4α
c Human hepatocytes Medium + Galectin-3
d Human hepatocytes Medium + CTGF
e Female, 46 years
P = 0.0418
Proliferating hepatocytes per well
150
100
50
0
Un-stretched
Stretched
Medium + Galectin-3
Medium + CTGF
f Human hepatocytes Unstretched
g Human hepatocytes Stretched
EdU HNF4α
h Human hepatocytes Medium + Galectin-3
i Human hepatocytes Medium + CTGF
j Male, 23 years
P = 0.0400
Proliferating hepatocytes per well
50
40
30
20
10
0
Un-stretched
Stretched
Medium + Galectin-3
Medium + CTGF

Supplementary Figure 3 (related to Figure 2): Galectin-3 and CTGF do not induce proliferation of primary human hepatocytes. a, b Representative laser scanning microscopy (LSM) images of human hepatocytes treated with the supernatant of unstretched (a) and stretched (b) hepatic endothelial cells (ECs). **c, d** Representative LSM images of human hepatocytes treated with hepatocyte maintenance medium plus galectin-3 (c) or connective tissue growth factor (CTGF, d). White arrowheads point to proliferating cells stained for 5-ethynyl-2'-deoxyuridine (EdU, green) and hepatocytes stained for hepatocyte nuclear factor 4α (HNF4α, red). **e** Quantification of proliferating hepatocytes from a female 46-year-old donor. n = 8 wells of human hepatocytes treated with the supernatant of unstretched or stretched hepatic ECs (used as control to demonstrate that human hepatocytes treated with the supernatant of stretched hepatic ECs show increased proliferation) and n = 4 wells treated with galectin-3 or CTGF. **f, g** Representative LSM images of human hepatocytes treated with the supernatant from unstretched (f) and stretched (g) hepatic ECs. **h, i** Representative LSM images of human hepatocytes treated with hepatocyte maintenance medium plus galectin-3 (h) or CTGF (i). White arrowheads point to proliferating cells stained for both, EdU (green) and HNF4α (red). **j** Quantification of proliferating hepatocytes in a male 23-year-old donor. n = 7 wells of human hepatocytes treated with the supernatant of unstretched hepatic ECs versus n = 8 wells treated with the supernatant of stretched hepatic ECs, and n = 4 wells treated with galectin-3 or CTGF. Scale bars: 50 µm (d, i). Data are presented as mean ± SEM. *P* values were calculated using one-way ANOVA followed by Dunnett's post hoc test. Source data are provided as a Source Data file.

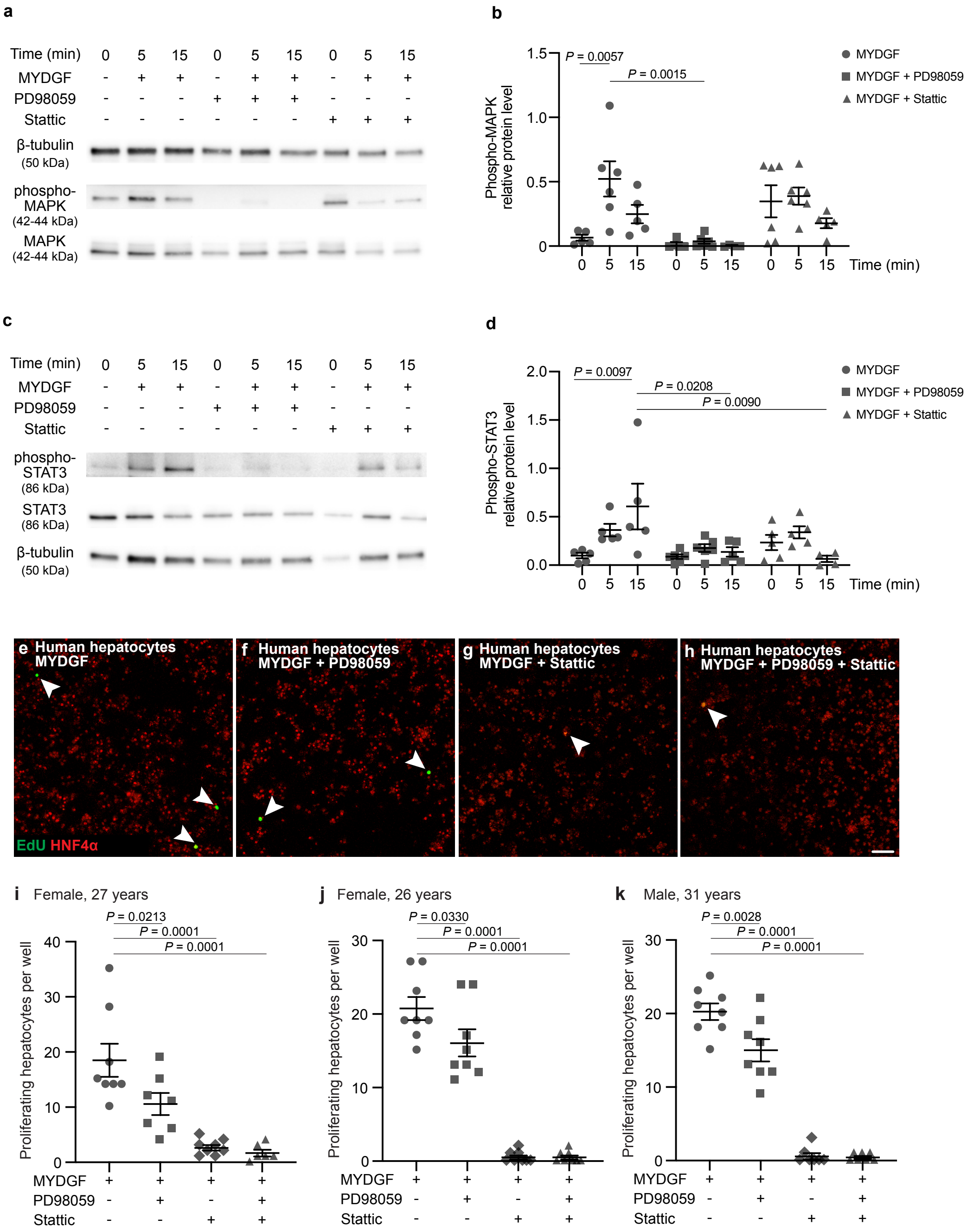

a
Time (min) 0 5 15 0 5 15 0 5 15
MYDGF - + + - + + - + +
PD98059 - - - + + + - - -
Stattic - - - - - - + + +
β-tubulin (50 kDa)
phospho-MAPK (42-44 kDa)
MAPK (42-44 kDa)
b
Phospho-MAPK relative protein level
P = 0.0057
P = 0.0015
MYDGF
MYDGF + PD98059
MYDGF + Stattic
Time (min)
c
Time (min) 0 5 15 0 5 15 0 5 15
MYDGF - + + - + + - + +
PD98059 - - - + + + - - -
Stattic - - - - - - + + +
phospho-STAT3 (86 kDa)
STAT3 (86 kDa)
β-tubulin (50 kDa)
d
Phospho-STAT3 relative protein level
P = 0.0097
P = 0.0208
P = 0.0090
MYDGF
MYDGF + PD98059
MYDGF + Stattic
Time (min)
e Human hepatocytes MYDGF
f Human hepatocytes MYDGF + PD98059
g Human hepatocytes MYDGF + Stattic
h Human hepatocytes MYDGF + PD98059 + Stattic
EdU HNF4α
i Female, 27 years
P = 0.0213
P = 0.0001
P = 0.0001
Proliferating hepatocytes per well
j Female, 26 years
P = 0.0330
P = 0.0001
P = 0.0001
k Male, 31 years
P = 0.0028
P = 0.0001
P = 0.0001
MYDGF + + + +
PD98059 - + - +
Stattic - - + +

Supplementary Figure 4 (related to Figure 3): Inhibition of phosphorylation of MAPK, STAT3 or both reduces human hepatocyte proliferation in the presence of MYDGF. a Phosphorylated mitogen-activated protein kinase (phospho-MAPK, 42-44 kDa; T202/Y204), MAPK (42-44 kDa) and β-tubulin (50 kDa) in lysates of human hepatocytes treated without or with MYDGF plus either PD98059 or Stattic. **b** Phospho-MAPK protein levels normalized to MAPK and β-tubulin. n = 5 (0, 15 min) and n = 6 (5 min) MYDGF; n = 5 (0 min), n = 6 (5 min) and n = 4 (15 min) MYDGF + PD98059; n = 6 (0, 5 min) and n = 5 (15 min) MYDGF + Stattic. **c** Phosphorylated signal transducer and activator of transcription 3 (phospho-STAT3, 86 kDa; S727), STAT3 (86 kDa) and β-tubulin (50 kDa) in lysates from human hepatocytes. **d** Phospho-STAT3 protein levels normalized to STAT3 and β-tubulin. n = 5 (0, 5, 15 min) MYDGF; n = 6 (0, 5 min) and n = 5 (15 min) MYDGF + PD98059; n = 5 (0, 5 min) and n = 4 (15 min) MYDGF + Stattic. **e-h** Laser scanning microscopy images of human hepatocytes treated with MYDGF (e), MYDGF plus PD98059 (f) or Stattic (g), or PD98059 and Stattic (h). Hepatocytes stained for 5-ethynyl-2'-deoxyuridine (EdU, green) and hepatocyte nuclear factor 4α (HNF4α, red). **i-k** Quantification of proliferating human hepatocytes. n = 8 (MYDGF), n = 7 (MYDGF + PD98059), n = 8 (MYDGF + Stattic), n = 6 (MYDGF + PD98059 + Stattic) wells of hepatocytes (i); n = 8 wells of hepatocytes each (j); n = 8 (MYDGF), n = 8 (MYDGF + PD98059), n = 7 (MYDGF + Stattic), n = 7 (MYDGF + PD98059 + Stattic) wells of hepatocytes (k). Donors: female, 26- (b, d, j) and 27-year-old (i); male, 31-year-old (k). Scale bar: 100 µm (h). Data are presented as mean ± SEM. *P* values were calculated using two-way ANOVA followed by Tukey´s post hoc test (b, d), and one-way ANOVA followed by Dunnett´s post hoc test (i-k). Source data are provided as a Source Data file.

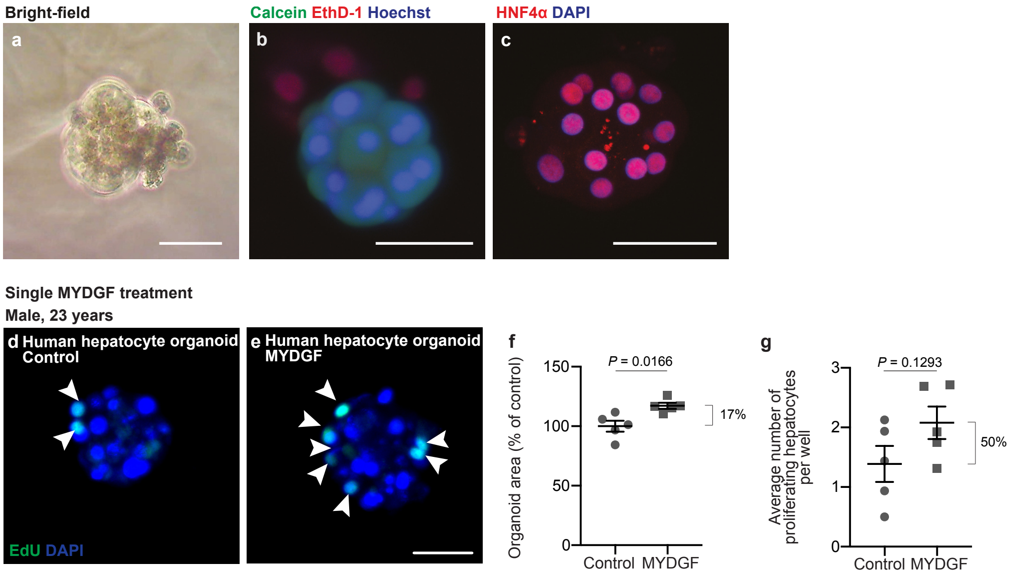
Bright-field
Calcein EthD-1 Hoechst
HNF4α DAPI
a
b
c
Single MYDGF treatment
Male, 23 years
d Human hepatocyte organoid
Control
e Human hepatocyte organoid
MYDGF
EdU DAPI
f
P = 0.0166
17%
Organoid area (% of control)
150
100
50
0
Control
MYDGF
g
P = 0.1293
50%
Average number of proliferating hepatocytes per well
3
2
1
0
Control
MYDGF

Supplementary Figure 5 (related to Figure 4): Primary human hepatocyte organoids. **a** Representative bright-field image of a growing human hepatocyte organoid in culture medium. **b** Representative laser scanning microscopy (LSM) image of a human hepatocyte organoid in a live-dead viability/cytotoxicity assay. Viable cells stained for calcein (green), dead cells stained for ethidium homodimer-1 (EthD-1, red), and cell nuclei stained for Hoechst (blue). **c** Representative LSM image of a human hepatocyte organoid in a whole mount staining. Hepatocytes were stained for hepatocyte nuclear factor 4α (HNF4α, red) and cell nuclei were counterstained for DAPI (blue). **d, e** Representative LSM images (maximum intensity projections) of human hepatocyte organoids treated without (d) or with (e) a single dose of recombinant MYDGF. Proliferating cells were stained for 5-ethynyl-2'-deoxyuridine (EdU, green) and cell nuclei counterstained for DAPI (blue). **f, g** Quantification of human organoid area (f) and proliferating human hepatocytes per well (g) in control- versus MYDGF-treated human hepatocyte organoids. Donor: male, 23 years; n = 5 wells with 6 organoids on average each. Scale bars: 100 µm (a) and 50 µm (b, c, e). Data are presented as mean ± SEM. *P* values were calculated using two-tailed unpaired Student's *t*-test with Welch's correction. Source data are provided as a Source Data file.

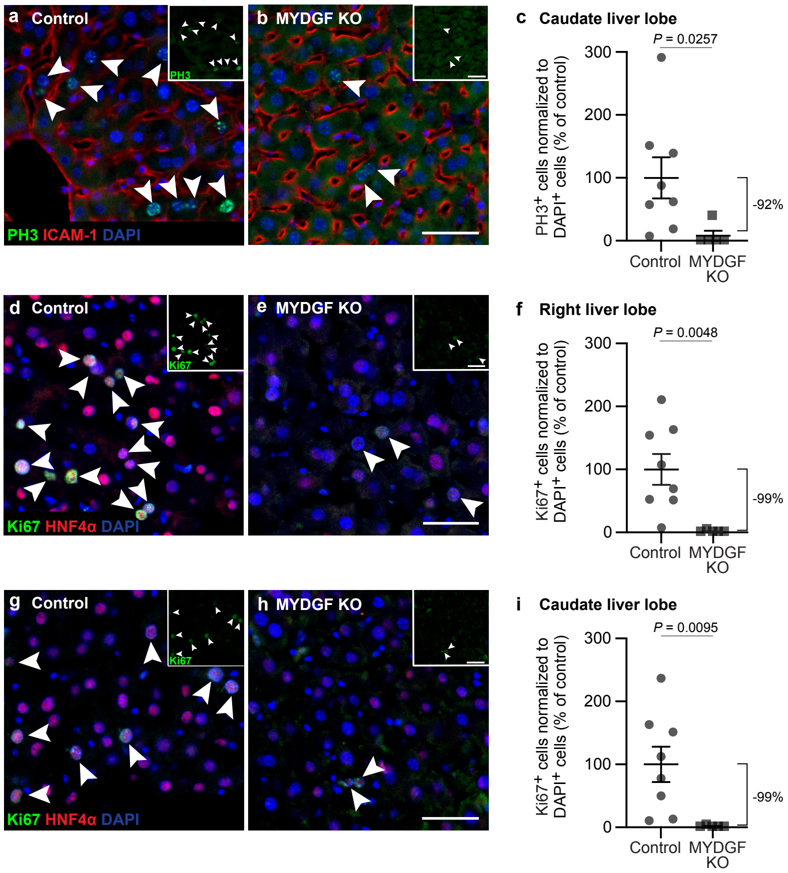
a Control
PH3
PH3 ICAM-1 DAPI
b MYDGF KO
c Caudate liver lobe
P = 0.0257
PH3+ cells normalized to DAPI+ cells (% of control)
300
200
100
0
-92%
Control
MYDGF KO
d Control
Ki67
Ki67 HNF4α DAPI
e MYDGF KO
f Right liver lobe
P = 0.0048
Ki67+ cells normalized to DAPI+ cells (% of control)
-99%
g Control
Ki67
Ki67 HNF4α DAPI
h MYDGF KO
i Caudate liver lobe
P = 0.0095
-99%

Supplementary Figure 6 (related to Figure 5): MYDGF KO in mice reduces hepatocyte proliferation in vivo after partial hepatectomy. a, b Representative laser scanning microscopy (LSM) images of the caudate liver lobe. Proliferating cells stained for phospho-Histone H3 (PH3, green, shown in insets, top right), blood vessels stained for intercellular adhesion molecule-1 (ICAM-1, red), and cell nuclei counterstained for DAPI (blue). White arrowheads point to $PH3^+$ cells. **c** Quantification of the number of $PH3^+$ cells normalized to the number of $DAPI^+$ cells in control versus MYDGF knockout (KO) mice, shown as percentage of control. **d, e** Representative LSM images of the right liver lobe. Proliferating cells stained for Ki67 (green, shown in insets, top right), hepatocytes stained for hepatocyte nuclear factor 4α (HNF4α, red), and cell nuclei counterstained for DAPI (blue). White arrowheads point to $Ki67^+$ cells. **f** Quantification of the number of $Ki67^+$ cells normalized to the number of $DAPI^+$ cells in control versus MYDGF KO mice, shown as percentage of control. **g, h** Representative LSM images of the caudate liver lobe. Proliferating cells stained for Ki67 (green, shown in insets, top right), hepatocytes stained for HNF4α (red), and cell nuclei counterstained for DAPI (blue). White arrowheads point to $Ki67^+$ cells. **i** Quantification of the number of $Ki67^+$ cells normalized to the number of $DAPI^+$ cells in control versus MYDGF KO mice, shown as percentage of control. n = 8 control versus n = 5 MYDGF KO transversal sections of the caudate (c, i) and right (f) liver lobe. Scale bars: 50 µm (b, e, h). Data are presented as mean ± SEM. *P* values were calculated using two-tailed unpaired Student's *t*-test with Welch's correction. Source data are provided as a Source Data file.

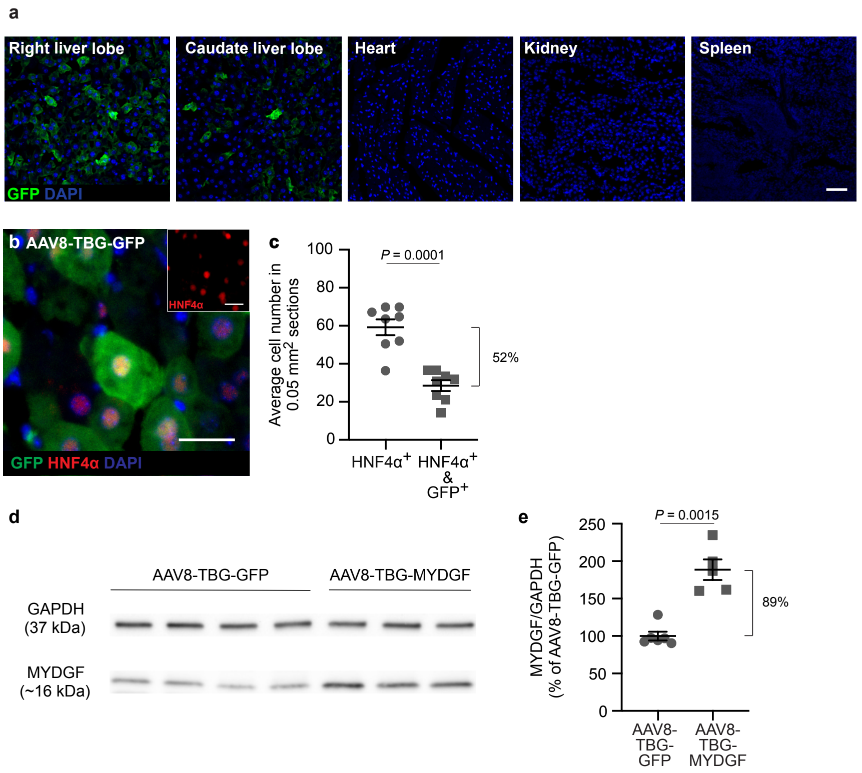
a
Right liver lobe
Caudate liver lobe
Heart
Kidney
Spleen
GFP DAPI
b AAV8-TBG-GFP
HNF4α
GFP HNF4α DAPI
c
Average cell number in 0.05 mm² sections
P = 0.0001
52%
HNF4α+
HNF4α+ & GFP+
d
AAV8-TBG-GFP
AAV8-TBG-MYDGF
GAPDH (37 kDa)
MYDGF (~16 kDa)
e
MYDGF/GAPDH (% of AAV8-TBG-GFP)
P = 0.0015
89%
AAV8-TBG-GFP
AAV8-TBG-MYDGF

Supplementary Figure 7 (related to Figure 5): AAV8-TBG-mediated expression of GFP or MYDGF in the liver. a Representative laser scanning microscopy (LSM) images of the right and caudate liver lobe, heart, kidney and spleen of mice transfected with an adeno-associated-virus of serotype 8 (AAV8) driving green fluorescent protein (GFP) under the control of a *thyroxine binding globulin* (TBG) promoter. Immunohistochemical GFP staining (green) visualized AAV8-TBG-mediated expression of GFP in liver cells. Cell nuclei were counterstained for DAPI (blue). **b** LSM image of the median mouse liver lobe stained for GFP (green), hepatocyte nuclear factor 4α (HNF4α, red, shown in inset, top right), and cell nuclei counterstained for DAPI (blue). **c** Quantification of the average number of $HNF4\alpha^+$ cells versus the average number of $HNF4\alpha^+$ and GFP^+ double-positive cells in 0.05 mm^2 sections of the median liver lobe. n = 8 AAV8-TBG-GFP transfected mice. **d** Representative Western blot images of liver lysates of AAV8-TBG-GFP- and AAV8-TBG-myeloid derived growth factor (MYDGF)-transfected mice showing MYDGF (~16 kDa) and GAPDH (37 kDa). **e** Quantification of MYDGF protein levels in liver lysates normalized to GAPDH protein levels, shown as percentage of MYDGF expressed in AAV8-TBG-GFP-transfected liver lysates; n = 6 AAV8-TBG-GFP versus n = 5 AAV8-TBG-MYDGF liver lysates. Scale bars: 50 µm (a), 25 µm (b). Data are presented as mean ± SEM. *P* values were calculated using two-tailed unpaired Student's *t*-test with Welch's correction. Source data are provided as a Source Data file.

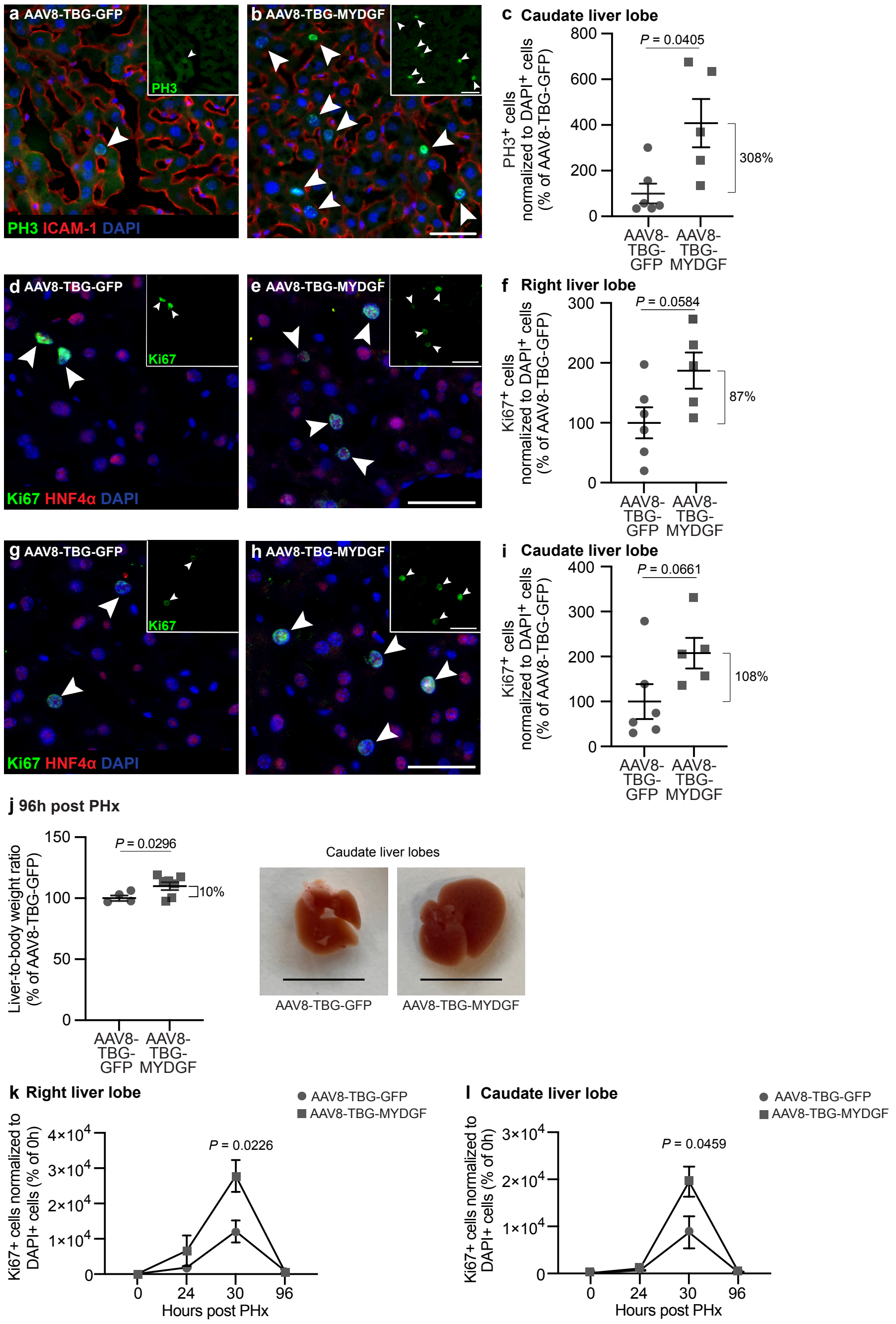
a AAV8-TBG-GFP
PH3
PH3 ICAM-1 DAPI
b AAV8-TBG-MYDGF
c Caudate liver lobe
PH3+ cells normalized to DAPI+ cells (% of AAV8-TBG-GFP)
P = 0.0405
308%
AAV8-TBG-GFP
AAV8-TBG-MYDGF
d AAV8-TBG-GFP
Ki67
Ki67 HNF4α DAPI
e AAV8-TBG-MYDGF
f Right liver lobe
Ki67+ cells normalized to DAPI+ cells (% of AAV8-TBG-GFP)
P = 0.0584
87%
g AAV8-TBG-GFP
h AAV8-TBG-MYDGF
i Caudate liver lobe
P = 0.0661
108%
j 96h post PHx
Liver-to-body weight ratio (% of AAV8-TBG-GFP)
P = 0.0296
10%
Caudate liver lobes
AAV8-TBG-GFP
AAV8-TBG-MYDGF
k Right liver lobe
Ki67+ cells normalized to DAPI+ cells (% of 0h)
P = 0.0226
Hours post PHx
l Caudate liver lobe
P = 0.0459

Supplementary Figure 8 (related to Figure 5): Growth-promoting effect of MYDGF on hepatocytes in mouse liver tissue after partial hepatectomy. a, b Laser scanning microscopy (LSM) images of the caudate liver lobe of mice transfected with an adeno-associated-virus of serotype 8 (AAV8) driving under the control of a *thyroxine binding globulin* (TBG) promoter, green fluorescent protein (GFP) or myeloid-derived growth factor (MYDGF) after partial hepatectomy (PHx). Phospho-Histone H3 (PH3, green), intercellular adhesion molecule-1 (ICAM-1, red) and DAPI (blue). **c** $PH3^+$ cells normalized to $DAPI^+$ cells in AAV8-TBG-GFP versus AAV8-TBG-MYDGF (percentage of AAV8-TBG-GFP). n = 6 AAV8-TBG-GFP versus n = 5 AAV8-TBG-MYDGF transversal sections of the caudate liver lobe. **d, e** LSM images of the right liver lobe of AAV8-TBG-GFP- and AAV8-TBG-MYDGF-transfected mice after PHx. Ki67 (green), hepatocyte nuclear factor 4α (HNF4α, red), and DAPI (blue). **f** $Ki67^+$ cells normalized to $DAPI^+$ cells in AAV8-TBG-GFP versus AAV8-TBG-MYDGF (percentage of AAV8-TBG-GFP). n = 6 AAV8-TBG-GFP versus n = 5 AAV8-TBG-MYDGF transversal sections of the right liver lobe. **g, h** LSM images of the caudate liver lobe of AAV8-TBG-GFP- and AAV8-TBG-MYDGF-transfected mice after PHx. Ki67 (green), HNF4α (red), and DAPI (blue). **i** $Ki67^+$ cells normalized to $DAPI^+$ cells in AAV8-TBG-GFP versus AAV8-TBG-MYDGF (percentage of AAV8-TBG-GFP). n = 6 AAV8-TBG-GFP versus n = 5 AAV8-TBG-MYDGF transversal sections of the caudate liver lobe. **j** Liver-to-body weight ratio of AAV8-TBG-GFP- and AAV8-TBG-MYDGF-transfected mice 96h after PHx with images of representative livers. n = 4 AAV8-TBG-GFP versus n = 7 AAV8-TBG-MYDGF. **k, l** $Ki67^+$ cells normalized to $DAPI^+$ cells in AAV8-TBG-GFP- versus AAV8-TBG-MYDGF-transfected mice. n = 4 (0 and 96h), n = 5 (24h), n = 6 (30h) AAV8-TBG-GFP versus n = 4 (0 and 24h), n = 5 (30h), n = 7 (96h) AAV8-TBG-MYDGF transversal sections of the right (k) and caudate (l) liver lobe each. Scale bars: 50 µm (b, e, h), 1 cm (j). Data are presented as mean ± SEM. *P* values were calculated using two-tailed unpaired Student's *t*-test with Welch's correction (c, f, i, j) or multiple unpaired *t*-test with Welch's correction (k, l). Source data are provided as a Source Data file.

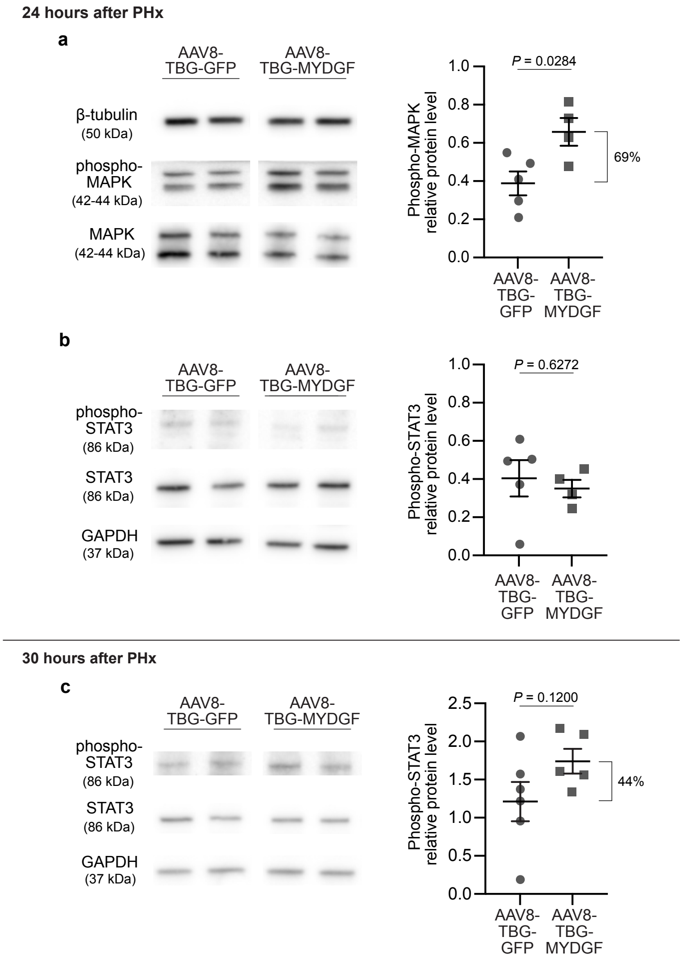
24 hours after PHx
a
AAV8-TBG-GFP
AAV8-TBG-MYDGF
β-tubulin
(50 kDa)
phospho-MAPK
(42-44 kDa)
MAPK
(42-44 kDa)
P = 0.0284
Phospho-MAPK relative protein level
69%
1.0
0.8
0.6
0.4
0.2
0.0
AAV8-TBG-GFP
AAV8-TBG-MYDGF
b
AAV8-TBG-GFP
AAV8-TBG-MYDGF
phospho-STAT3
(86 kDa)
STAT3
(86 kDa)
GAPDH
(37 kDa)
P = 0.6272
Phospho-STAT3 relative protein level
1.0
0.8
0.6
0.4
0.2
0.0
AAV8-TBG-GFP
AAV8-TBG-MYDGF
30 hours after PHx
c
AAV8-TBG-GFP
AAV8-TBG-MYDGF
phospho-STAT3
(86 kDa)
STAT3
(86 kDa)
GAPDH
(37 kDa)
P = 0.1200
Phospho-STAT3 relative protein level
44%
2.5
2.0
1.5
1.0
0.5
0.0
AAV8-TBG-GFP
AAV8-TBG-MYDGF

Supplementary Figure 9 (related to Figure 5): MYDGF activates phosphorylation of MAPK in regenerating mouse livers after partial hepatectomy in vivo. a Representative Western blots of the right liver lobe of mice transfected with an adeno-associated-virus of serotype 8 (AAV8) driving under the control of a *thyroxine binding globulin* (TBG) promoter, green fluorescent protein (GFP) or myeloid-derived growth factor (MYDGF), 24h after partial hepatectomy (PHx). Phosphorylation of mitogen-activated protein kinase (MAPK, 42-44 kDa; T202/Y204), MAPK (42-44 kDa) and β-tubulin (50 kDa), and quantification of phospho-MAPK protein levels normalized to MAPK and β-tubulin protein levels. **b** green fluorescent protein (GFP)**, c** Representative Western blot images of the right liver lobe of mice 24h (b) and 30h (c) after PHx showing phosphorylation of signal transducer and activator of transcription 3 (STAT3, 86 kDa; S727), STAT3 (86 kDa) and GAPDH (37 kDa), and quantification of phospho-STAT3 protein levels normalized to STAT3 and GAPDH protein levels. n = 5 AAV8-TBG-GFP versus n = 4 AAV8-TBG-MYDGF liver lysates 24h post PHx (a, b) and n = 6 AAV8-TBG-GFP versus n = 5 AAV8-TBG-MYDGF liver lysates 30h post PHx (c). Data are presented as mean ± SEM. *P* values were calculated using two-tailed unpaired Student’s *t*-test with Welch’s correction. Source data are provided as a Source Data file.
