# Supplementary material for: Identification of myeloid-derived growth factor as a mechanically-induced, growth-promoting angiocrine signal for human hepatocytes
Source: Nat Commun. 2024 Feb 5;15:1076. doi: 10.1038/s41467-024-44760-y (PMC10844291; doi:10.1038/s41467-024-44760-y)
Supplement: Supplementary file 2 — Reporting Summary [file 41467_2024_44760_MOESM2_ESM.pdf]

nature portfolio

Corresponding author(s): Eckhard Lammert

Last updated by author(s): Dec 18, 2023

Reporting Summary

Nature Portfolio wishes to improve the reproducibility of the work that we publish. This form provides structure for consistency and transparency in reporting. For further information on Nature Portfolio policies, see our Editorial Policies and the Editorial Policy Checklist.

Statistics

For all statistical analyses, confirm that the following items are present in the figure legend, table legend, main text, or Methods section.

n/a	Confirmed	
☐	☒	The exact sample size (*n*) for each experimental group/condition, given as a discrete number and unit of measurement
☐	☒	A statement on whether measurements were taken from distinct samples or whether the same sample was measured repeatedly
☐	☒	The statistical test(s) used AND whether they are one- or two-sided<br>*Only common tests should be described solely by name; describe more complex techniques in the Methods section.*
☐	☒	A description of all covariates tested
☐	☒	A description of any assumptions or corrections, such as tests of normality and adjustment for multiple comparisons
☐	☒	A full description of the statistical parameters including central tendency (e.g. means) or other basic estimates (e.g. regression coefficient) AND variation (e.g. standard deviation) or associated estimates of uncertainty (e.g. confidence intervals)
☐	☒	For null hypothesis testing, the test statistic (e.g. *F*, *t*, *r*) with confidence intervals, effect sizes, degrees of freedom and *P* value noted<br>*Give P values as exact values whenever suitable.*
☒	☐	For Bayesian analysis, information on the choice of priors and Markov chain Monte Carlo settings
☒	☐	For hierarchical and complex designs, identification of the appropriate level for tests and full reporting of outcomes
☒	☐	Estimates of effect sizes (e.g. Cohen's *d*, Pearson's *r*), indicating how they were calculated

Our web collection on statistics for biologists contains articles on many of the points above.

Software and code

Policy information about availability of computer code

Data collection	Image Lab Touch Software Version 2.4.0.03 (Bio-Rad) was used for Western Blots, LightCycler® Nano Software 1.1 (Roche) and QuantStudioTM Design & Analysis Software v1.5.1 (Thermo Fisher Scientific) were used for qPCR , GloMax® Discover Version 3.2.3 (Promega GmbH) was used for ELISAs and BCA-Assays, CytExpert Version 2.4.0.28 (Beckman Coulter) was used for flow cytometry and Zen 2.3 SP1 FP3 black (Zeiss) was used for image acquisition.
Data analysis	Western blots were analyzed using Image Lab Software Version 5.2 and 6.1 (Bio-Rad). FlowJo software version 10 (BD Biosciences, RRID:SCR_008520) was used for the quantification of the flow cytometric experiment. FIJI (ImageJ, NIH) and QuPath (Open source software for digital pathology image analysis) were used for all analyses of microscopy data. Prism version 10 (GraphPad) and Excel version 16.77 (Microsoft) were used for statistical analyses.

For manuscripts utilizing custom algorithms or software that are central to the research but not yet described in published literature, software must be made available to editors and reviewers. We strongly encourage code deposition in a community repository (e.g. GitHub). See the Nature Portfolio guidelines for submitting code & software for further information.

Data

Policy information about availability of data

All manuscripts must include a data availability statement. This statement should provide the following information, where applicable:

- Accession codes, unique identifiers, or web links for publicly available datasets
- A description of any restrictions on data availability
- For clinical datasets or third party data, please ensure that the statement adheres to our policy

Source data for all quantifications are provided. The mass spectrometry proteomics data have been deposited to the ProteomeXchange Consortium via the PRIDE partner repository with the dataset identifier PXD033942. Gating criteria of flow cytometric analysis and full scans of western blots are provided in Source Data file.

Research involving human participants, their data, or biological material

Policy information about studies with human participants or human data. See also policy information about sex, gender (identity/presentation), and sexual orientation and race, ethnicity and racism.

Reporting on sex and gender	Patients are included consecutively without regard to sex and gender.
Reporting on race, ethnicity, or other socially relevant groupings	Patients are included consecutively without regard to ethnicity,race or individuals belonging to a certain social group.
Population characteristics	Human blood serum were obtained from one patient with in-situ split liver resection and human blood plasma was obtained from six patients with liver resection (Fig. 1i,j). A brief summary of patient characteristic is provided in the Methods section: "In-situ split liver resection and liver resection".
Recruitment	Not relevant for these patients. For Fig. 1j, we excluded individuals with obesity (BMI > 30 kg/m2).
Ethics oversight	Fig 1i: This study was approved by the local institutional review board (Heinrich Heine University, Duesseldorf, Germany; 2018–258-KFogU). Fig. 1j: The study was positively evaluated by the Ethics Committee of the Landesärztekammer Rheinland-Pfalz (2020-15149).

Note that full information on the approval of the study protocol must also be provided in the manuscript.

Field-specific reporting

Please select the one below that is the best fit for your research. If you are not sure, read the appropriate sections before making your selection.

☒ Life sciences ☐ Behavioural & social sciences ☐ Ecological, evolutionary & environmental sciences

For a reference copy of the document with all sections, see nature.com/documents/nr-reporting-summary-flat.pdf

Life sciences study design

All studies must disclose on these points even when the disclosure is negative.

Sample size	In vivo: Sample size was calculated for control and MYDGF KO mice with Power Calculator 2 and for AAV8-TBG-GFP and AAV8-TBG-MYDGF mice with G*Power Version 3.1.9.3. In vitro: No sample-size calculation was performed (we used different donors of hepatocytes, each containing a different number of cells). Experiments were generally conducted with at least n = 4 biological samples. The precise number (n) of biological samples used to derive statistics is indicated in the figure legends.
Data exclusions	For in vivo experiments, mice with improperly perfused (light brown) livers and hemorrhages in the livers were excluded from further analysis. For Western blots, bands with air bubbles were excluded from further analysis. Significant outliers were detected by ESD method (Grubbs' test) in PRISM and excluded from analysis.
Replication	Stretch experiments with human hepatic ECs (shown in Fig. 1b-d). EdU proliferation assay (shown in Fig. 2a-f and Supplementary Fig. 2a-c) and PH3 staining (shown in Fig. 2g-i and Supplementary Fig. 2d-f) in 2D cultured human hepatocytes. TUNEL staining (shown in Fig. 2j-l and Supplementary Fig. 2k-n) and caspase-3 staining (shown in Fig. 2m-o and Supplementary Fig. 2o-q) in 2D cultured human hepatocytes. Western blots for phospho-MAPK and phospho-STAT3 (Fig. 3a-d) were replicated with 100 ng/ml MYDGF (data not shown). EdU proliferation assay in 3D cultured human hepatocytes (shown in Fig. 4b-i and Supplementary Fig. 5d-f). PH3 staining in control versus MYDGF KO mice (shown in Fig. 5e-g and Supplementary Fig. 6a-c) was reproduced with an additional proliferation marker, i.e., Ki67 (shown in Supplementary Fig. 6d-i). PH3 staining in AAV8-TBG-GFP versus AAV8-TBG-MYDGF mice (shown in Fig. 5i-k and Supplementary Fig. 8a-c) was reproduced with an additional proliferation marker, i.e., Ki67 (shown in Supplementary Fig. 8d-i). PH3 staining of HepG2 cells (Supplementary Fig. 2g-i) was replicated with 100 ng/ml MYDGF (data not shown).
Randomization	For AAV8-TBG-GFP or AAV8-TBG-MYDGF injection, mice were randomized into the respective groups. For 2D in vitro experiments with primary human hepatocytes and EdU proliferation assay with 3D human hepatocyte organoids, a lab member (independent of the experiment) randomly selected which hepatocytes received which treatment.

Blinding	The two-thirds partial hepatectomy of control and MYDGF KO mice or AAV8-TBG-GFP and AAV8-TBG-MYDGF mice was performed in a blinded fashion, i.e. the operators did not know which animal of which group they were operating on. 2D in vitro experiments with primary human hepatocytes and the EdU proliferation assay with 3D human hepatocyte organoids were performed and analyzed in a blinded manner. All microscopy images were blinded and some of them were analyzed in an automated fashion.

Reporting for specific materials, systems and methods

We require information from authors about some types of materials, experimental systems and methods used in many studies. Here, indicate whether each material, system or method listed is relevant to your study. If you are not sure if a list item applies to your research, read the appropriate section before selecting a response.

Materials & experimental systems

n/a	Involved in the study	
☐	☒	Antibodies
☐	☒	Eukaryotic cell lines
☒	☐	Palaeontology and archaeology
☐	☒	Animals and other organisms
☒	☐	Clinical data
☒	☐	Dual use research of concern
☒	☐	Plants

Methods

n/a	Involved in the study	
☒	☐	ChIP-seq
☐	☒	Flow cytometry
☒	☐	MRI-based neuroimaging

Antibodies

Antibodies used	For immunostaining the following primary antibodies were used: rabbit anti-phospho-Histone H3 (Ser10) (Sigma-Aldrich, 06-570, Lot: 3795233, 1/50); rabbit anti-caspase-3 (Sigma-Aldrich, C8487, Lot: 059K4750, 1/200); rabbit anti-HNF4α (CellSignaling, C11F12, 3113, Lot: 4, 1/500), goat anti-ICAM-1/CD54 (R&D, AF796, Lot: GRG0220081, 1/100); rat anti-Ki-67 (SolA15) (Invitrogen, 14-5698-82, Lot: 2355034, 1/100), rabbit anti-HNF4α (Abcam, ab181604, Lot: 1026268-5, 1/100), goat anti-GFP (Sicgen, AB0020-200, Lot: 0020190219, 1/500). Secondary antibodies were used as follows: donkey anti-rabbit/rat/goat Alexa Fluor 488 (Invitrogen, A-21206, Lot: 2330673/ A-21208, Lot: 2482958/ A-11055, Lot: 2301114; 1/500), donkey anti-goat/ rabbit Alexa Fluor 555 (Invitrogen, A-21432, Lot: 2540864/ A-31572, Lot: 2339822; 1/500), and DAPI (Sigma-Aldrich, D9542, 1/1000). For MACS anti-CD146 mouse MicroBeads (Miltenyi Biotec, 130-092-007) were used. For Western blotting following antibodies were used: rabbit anti-MYDGF (Proteintech, 11353-1-AP, Lot: 00064724, 1/1000), rabbit anti-Phospho-p44/42 MAPK (Erk1/2) (Thr202/Tyr204) (Cell Signaling, 4376, Lot: 21, 1/750 or 1/500), rabbit anti-p44/42 MAPK (Erk1/2) (137F5) (Cell Signaling, 4695, Lot: 28, 1/750 or 1/500), rabbit anti-Phospho-Stat3 (Ser727) (Cell Signaling, 9134, Lot: 21, 1/500), rabbit anti-Stat3 (D3Z2G) (Cell Signaling, 12640, Lot: 7, 1/750), rabbit anti-Phospho-AKT (Ser473) (D9E) (Cell Signaling, 4060, Lot: 27, 1/750), rabbit anti-AKT (pan) (C67E7) (Cell Signaling, 4691, Lot: 20, 1/750), rabbit anti-GAPDH (Abcam, ab9485, Lot: GR3437709-1, 1/5000 or 1/2000) and rabbit anti-β tubulin antibody (Abcam, ab6046, Lot: GR3441353-1, 1/2000), goat anti rabbit IgG, HRP-linked (Cell Signaling, 7074, Lot: 27, 1/2000).
Validation	Antibodies were validated by the suppliers as follows: rabbit anti-phospho-Histone H3 (Ser10) (Sigma-Aldrich, 06-570): Immunohistochemistry (IHC), Western Blot (WB), Immunocytochemistry (ICC); rabbit anti-caspase-3 (Sigma-Aldrich, C8487): Immunofluorescent (IF), WB; rabbit anti-HNF4α (Cell Signaling, C11F12): WB, IHC,IF; goat anti-ICAM-1/CD54 (R&D, AF796): WB, IHC, adhesion blockade; rat anti-Ki-67 (SolA15) (Invitrogen, 14-5698-82): WB, IHC, ICC, Flow Cytomentry (Flow), Functional Assay (FN); rabbit anti-HNF4α (Abcam, ab181604): Chromatin Immuno-Cleavage/Cleavage Under Targets and Release Using Nuclease (ChIC/ CUT&RUN); chromatin immunoprecipitation (ChIP), IHC, WB, Immunoprecipitation (IP); goat anti-GFP (Sicgen, AB0020-200): WB, IF, IHC; donkey anti-rabbit Alexa Fluor 488 (Invitrogen, A-21206): IHC, ICC/IF, Flow; donkey anti-rat Alexa Fluor 488 (Invitrogen, A-21208): IHC, ICC/IF; donkey anti-goat Alexa Fluor 488 (Invitrogen, A-11055): IHC, ICC/IF, Flow; donkey anti-goat Alexa Fluor 555 (Invitrogen, A-21432): IHC, ICC/IF; donkey anti-rabbit Alexa Fluor 555 (Invitrogen, A-31572): IHC, ICC/IF, Flow; DAPI (Sigma-Aldrich, D9542): IF; rabbit anti-MYDGF (Proteintech, 11353-1-AP): WB (antibody was also validated in our laboratory by adding recombinant MYDGF protein (Novoprotein, CG64) on WB), IHC, IF, enzyme-linked immunosorbent assay (ELISA); rabbit anti-Phospho-p44/42 MAPK (Erk1/2) (Thr202/Tyr204) (Cell Signaling, 4376): WB, IP, IHC; rabbit anti-p44/42 MAPK (Erk1/2) (137F5) (Cell Signaling, 4695): WB, IP, IHC, IF, Flow; rabbit anti-Phospho-Stat3 (Ser727) (Cell Signaling, 9134): WB, IP, ChIP; rabbit anti-Stat3 (D3Z2G) (Cell Signaling, 12640): WB, IP, IF, Flow, ChIP; rabbit anti-Phospho-AKT (Ser473) (D9E) (Cell Signaling, 4060): WB, IP, IHC, IF, Flow; rabbit anti-AKT (pan) (C67E7) (Cell Signaling, 4691): WB, IP, IHC, IF, Flow; rabbit anti-GAPDH (Abcam, ab9485): IHC, WB, ICC/IF; rabbit anti-β tubulin antibody (Abcam, ab6046): WB, ICC/IF, IHC, IP; goat anti rabbit IgG, HRP-linked (Cell Signaling, 7074): WB.

Eukaryotic cell lines

Policy information about cell lines and Sex and Gender in Research

Cell line source(s)	HepG2 cells were purchased from ATCC (ATCC®HB-8065TM). Culture conditions are stated in the Methods section. HepG2 is a cell line that was isolated from a hepatocellular carcinoma from a 15-year-old male with liver cancer.
Authentication	HepG2 cells were not authenticated.
Mycoplasma contamination	HepG2 cells were mycoplasm negative (Mycoplasma check was performed by Eurofins).

Commonly misidentified lines (See ICLAC register)	No commonly misidentified cell line were used.

Animals and other research organisms

Policy information about studies involving animals; ARRIVE guidelines recommended for reporting animal research, and Sex and Gender in Research

Laboratory animals	C57BL/6N mice (Charles River), C57BL/6J mice (Janvier) and MYDGF KO (published in Korf-Klingebiel et al., Nat. Med. 2015) mice at 10-15 weeks of age were used. Details about the experimental procedures are provided in the methods section.
Wild animals	The study did not involve wild animals.
Reporting on sex	Male mice were used as indicated in the manuscript.
Field-collected samples	The study did not involve samples collected from the field.
Ethics oversight	All experiments were performed according to the German animal protection laws (Animal Ethics Committee of the Landesamt für Natur, Umwelt und Verbraucherschutz, Nordrhein-Westfalen).

Note that full information on the approval of the study protocol must also be provided in the manuscript.

Flow Cytometry

Plots

Confirm that:

- [x] The axis labels state the marker and fluorochrome used (e.g. CD4-FITC).
- [x] The axis scales are clearly visible. Include numbers along axes only for bottom left plot of group (a 'group' is an analysis of identical markers).
- [ ] All plots are contour plots with outliers or pseudocolor plots.
- [x] A numerical value for number of cells or percentage (with statistics) is provided.

Methodology

Sample preparation	Sample preparation is provided in the Methods section.
Instrument	Flow cytometry measurements were performed on CytoFlex S Flow Cytometer (Beckman Coulter, BE35105)
Software	Data was acquired using CytExpert 2.4.0.28 and analyzed using FlowJo V10.9.0.
Cell population abundance	A total of 10,000 events were acquired.
Gating strategy	Cells were gated from debris using an FSC-H/SSC-H contour plot. Subsequently, single cells were gated from cell clusters by gating the main population of an FSC-H/FSC-A plot. The histogram plots of the FVS660 (APC channel) staining were created from the single cell gate.

- [x] Tick this box to confirm that a figure exemplifying the gating strategy is provided in the Supplementary Information.
